# A molecular framework for autistic experiences: Mitochondrial allostatic load as a mediator between autism and psychopathology

**DOI:** 10.3389/fpsyt.2022.985713

**Published:** 2022-11-25

**Authors:** Caitlyn Mahony, Colleen O'Ryan

**Affiliations:** Department of Molecular and Cell Biology, University of Cape Town, Cape Town, South Africa

**Keywords:** autism (ASD), autistic burnout, social camouflaging, psychopathology, suicidality, early life stress (ELS), chronic adolescent stress (CAS), mitochondrial allostatic load

## Abstract

Molecular autism research is evolving toward a biopsychosocial framework that is more informed by autistic experiences. In this context, research aims are moving away from correcting external autistic behaviors and toward alleviating internal distress. Autism Spectrum Conditions (ASCs) are associated with high rates of depression, suicidality and other comorbid psychopathologies, but this relationship is poorly understood. Here, we integrate emerging characterizations of internal autistic experiences within a molecular framework to yield insight into the prevalence of psychopathology in ASC. We demonstrate that descriptions of social camouflaging and autistic burnout resonate closely with the accepted definitions for early life stress (ELS) and chronic adolescent stress (CAS). We propose that social camouflaging could be considered a distinct form of CAS that contributes to allostatic overload, culminating in a pathophysiological state that is experienced as autistic burnout. Autistic burnout is thought to contribute to psychopathology *via* psychological and physiological mechanisms, but these remain largely unexplored by molecular researchers. Building on converging fields in molecular neuroscience, we discuss the substantial evidence implicating mitochondrial dysfunction in ASC to propose a novel role for mitochondrial allostatic load in the relationship between autism and psychopathology. An interplay between mitochondrial, neuroimmune and neuroendocrine signaling is increasingly implicated in stress-related psychopathologies, and these molecular players are also associated with neurodevelopmental, neurophysiological and neurochemical aspects of ASC. Together, this suggests an increased exposure and underlying molecular susceptibility to ELS that increases the risk of psychopathology in ASC. This article describes an integrative framework shaped by autistic experiences that highlights novel avenues for molecular research into mechanisms that directly affect the quality of life and wellbeing of autistic individuals. Moreover, this framework emphasizes the need for increased access to diagnoses, accommodations, and resources to improve mental health outcomes in autism.

## Graphical Abstract

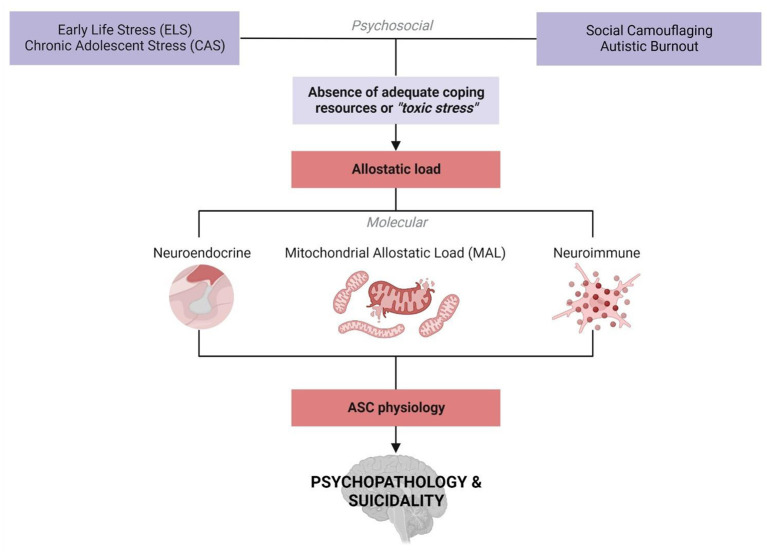

## Introduction

Research into autism spectrum conditions (ASCs) spans disparate psychosocial and biomedical disciplines that traditionally work in isolation. However, the complex and rapidly evolving sociological context of autism research calls for a more integrative approach. Psychosocial and clinical conceptualizations of ASC fundamentally define the parameters of molecular autism research. Therefore, it is essential to integrate evolving understandings of autistic experiences within a molecular framework. Historically, ASC has been defined by the external perceptions of neurotypical observers rather than an understanding of internal autistic experiences (1). This has manifested as an over-reliance on the medical model of disability in autism research, which was often rooted in the pathologization of ASC-associated behaviors. With the increasing incorporation of inclusive participatory research practices, the field is progressing toward a more comprehensive integration of the biopsychosocial disability model (2, 3). This interdisciplinary model recognizes that while the biological (4–7) and behavioral (8–11) differences associated with ASC present challenges to independent functioning, the inability to function within a societal framework that accommodates only one neurotype is not indicative of an underlying biological deficit (12, 13). Congruent with this, psychosocial research is shifting its focus to improving quality of life and minimizing internal distress, rather than eradicating or correcting autistic behaviors (14).

However, this paradigm shift is yet to be fully integrated into the medical and molecular fields of autism research, since these disciplines require unambiguous definitions of disease, reductionist animal and cell culture models, and highly specific questions with limited scope. The challenges of biomedical research are already compounded by the etiological heterogeneity of ASC (15, 16) and the limitations of its conceptualization in diagnostic texts (1, 17). These limitations are increasingly well-recognized in clinical settings, especially with respect to the questionable reliability of autism assessments in females and people of color across varied sociocultural settings (18–25). Yet, molecular studies require deeply-phenotyped cohorts characterized according to standardized diagnostic criteria in order to accurately distinguish between complex molecular signatures (26). In this context, an over-reliance on an antiquated deficits-based disability model is often unavoidable. Moreover, biomedical research often relies on limited measurements of behavior and flawed indicators of functioning. The efficacy of an intervention is frequently measured by a decrease in ASC diagnostic criteria, but it is becoming clear that this is not a good indicator of health, wellbeing or quality of life for autistic individuals. In fact, the chronic suppression of autistic traits contributes to anxiety, depression, burnout, a diminished quality of life and poor mental health in ASC (27–30). It is now recognized that therapeutic interventions should prioritize physical and psychological wellbeing, which may indeed manifest differently in different neurotypes. Of course, the association between internal distress and the external presentation of autistic behaviors will reflect the variability that is inherent to all facets of ASC etiology. Molecular interventions that target behavioral or biological differences may potentially improve health or wellbeing. Still, it should be acknowledged that much biomedical research has sought to decrease the “autism phenotype” without recognizing that this is not synonymous with an enhanced quality of life.

Nevertheless, molecular research has demonstrated potential to be relevant, useful and transformative for the autistic community. Genetic insight into the heritability of ASC has been instrumental in improving access to early diagnoses, and shifting blame away from caregivers or autistic individuals themselves for their differences. Moreover, an understanding of molecular ASC etiology has been used to mitigate common comorbidities that reduce quality of life. For example, molecular therapies have been developed to treat tumors and epilepsy in individuals with a monogenic form of autism called tuberous sclerosis (31). Concurrently, clinicians are still struggling with existing diagnostic guidelines that do not easily distinguish between ASC and co-morbidities, or reflect the heterogeneity in autism presentations, internal lived experiences or underlying biological processes. In conjunction with other psychosocial models, molecular research could be a useful tool to improve the resolution, specificity and utility of diagnostic labels. Ultimately, this could lead to a better understanding of individual autism presentations, their specific challenges, and relevant accommodations. Therefore, it is essential that molecular researchers grapple with the task of integrating the rigor of scientific experimental design and evolving understandings of autism within a biopsychosocial framework.

Importantly, an interdisciplinary approach could also yield novel mechanistic insights into molecular ASC etiology. Many promising findings from *in vitro* and *in vivo* molecular studies have failed to translate into reproducible results in human clinical trials (16, 32–36). This has largely been attributed to the complex interplay among genetic, epigenetic and environmental mechanisms in ASC. However, molecular research is also inherently reductionist and grounded in a one-dimensional understanding of ASC that is removed from the internal experience of autism. This could be a prevailing limitation that hinders the translatability of molecular research in a clinical and sociological context (1). Consequently, an integrative approach is needed to highlight the convergence between different scientific disciplines involved in autism research. In particular, this approach could provide crucial insight into the cellular mechanisms at the intersection of environmental stressors and biological processes that directly impact quality of life.

Here, we integrate recent characterizations of internal autistic experiences within a molecular framework to yield insight into psychopathology and suicidality in ASC. Interactions between psychosocial stress, ASC physiology and allostatic load have previously been proposed to influence clinical outcomes in autism (37–39). Singletary (37) proposed that autistic children are exposed to significant stress that could disrupt social and cognitive development during the early post-natal period. Thus, he suggested that interactions between early life stress (ELS), underlying physiology and allostatic overload may contribute to ASC presentations (37). More recently, Scarpa et al. (38) developed a biosocial vulnerability model which suggests that ASC neurophysiology mediates a predisposition to pathological trauma responses. However, this model remains understudied by molecular researchers.

In this article, we assimilate the emerging definitions for social camouflaging and autistic burnout with recent insights into the biological mechanisms that mediate the stress response. Previous authors focused on the relationship between ELS and the manifestation of autistic traits. However, we consider the putative role of ELS in mediating the relationship between ASC and psychopathology from a molecular perspective. Consequently, we propose an increased risk of exposure to, and an underlying molecular susceptibility to, ELS and Chronic Adolescent Stress (CAS) in ASC. Both ELS and CAS are known to contribute to psychopathology and a molecular interplay between mitochondrial allostatic load (MAL), the Hypothalamic-Pituitary-Adrenal (HPA) axis, and neuroinflammation is increasingly implicated in this association (40). Notably, mitochondrial dysfunction and neuroinflammation are widely documented molecular signatures in ASC, and each are known to regulate HPA axis signaling. Moreover, these signaling pathways converge on neurodevelopmental, neurophysiological and neurochemical processes that are disrupted in response to ELS and may be involved in the etiology of both ASC and psychopathology. This article describes an integrative framework shaped by autistic experiences that demonstrates how psychosocial and biological factors converge to increase the risk of psychopathology. This framework emphasizes the need to improve access to diagnoses, accommodations, and resources to mitigate poor mental health outcomes in autism. Moreover, this highlights novel avenues for future research into molecular mechanisms that affect the health and wellbeing of autistic individuals.

## Psychopathology and suicidality in ASC: The role of social camouflaging and autistic burnout

ASC is associated with a high prevalence of comorbid psychopathologies that substantially impair functioning and decrease quality of life (41–43). In particular, the rates of generalized anxiety disorder (GAD), major depressive disorder (MDD), post-traumatic stress disorder (PTSD), self-harm, and suicidal ideation are significantly increased in ASC (30, 44, 45). It was recently reported that autistic children between 10 and 13 years old were six times more likely to reach clinically significant thresholds for measures of depression (46), indicating an earlier onset of MDD in ASC. Moreover, three measures of suicidality are more than twice as high in autistic individuals compared to the general population—including the rates of suicide risk, suicide attempts, and mortality by self-harm and suicide (43, 44, 47, 48). Compounding this, there are unique challenges associated with the assessment of suicidal ideation and suicide risk given that alexithymia and communication difficulties are core components of ASC (49). The high prevalence of psychopathology in autism is currently attributed to social difficulties, alexithymia, cognitive rigidity, loneliness, a lack of social support and increased exposure to traumatic experiences (44, 45, 50, 51). In addition, it has been suggested that underlying genetic (52) and neurological (38, 46, 53) mechanisms in ASC etiology could mediate a susceptibility to depression and other mood disorders. Thus, it is critical to understand the internal experiences and physiological mechanisms that mediate the relationship between ASC and psychopathology.

Emerging research has begun to characterize the distinct phenomena known as autistic burnout and social camouflaging, based on the lived experiences of autistic individuals. Importantly, this nascent research has intrinsic limitations in that it does not yet represent the diversity of experiences across the autism spectrum (29). Ongoing research is still defining, operationalizing, and empirically validating the afore-mentioned constructs in a clinical and research context (54–58). Nevertheless, considering some internal experiences of ASC may yield valuable insights beyond the inferences based solely on observable behaviors. Moreover, autistic burnout and social camouflaging are both understudied, yet integral aspects of the internal experience of autism that could play a central role in distinguishing psychological health from psychopathology.

While the field is still refining a consensus definition for autistic burnout, complementary working definitions are converging on similar experiences. Autistic burnout has been described as “*a state of physiological and psychological incapacitation, exhaustion, and distress in every area of life*” (59) marked by loss of function, reduced tolerance to stimulus and an increased manifestation of autistic traits (60). This condition results from “*a mismatch of expectations and abilities without adequate supports*” (59) and directly impacts “*mental health, quality of life and well-being*” (61). Autistic burnout is conceptually similar to non-autistic burnout in that it results from chronically operating beyond personal capacity. However, autistic burnout is distinct with respect to its causes, manifestations and functional implications (62).

The main contributor to autistic burnout is social camouflaging, defined as “*the need to suppress autistic traits or disability… in order to meet family, social, vocational, or other mainstream expectations*” (59). While several different tools are emerging to quantitatively measure social camouflaging (55, 56, 58), current models agree on the presence of three core components: (i) deliberately adopting new social behaviors to make up for social difficulties (compensation); (ii) consciously suppressing autistic traits in order to appear non-autistic (masking); and (iii) implementing strategies to manage internal discomfort invisibly (assimilation) (22, 27, 58). Chronic social camouflaging leads to internal distress, an eroded sense of self, and physical and mental exhaustion (27, 63, 64) and is associated with psychopathology (29), self-injury (65–67), and a higher risk of lifetime suicidality (30).

However, camouflaging also facilitates access to employment, relationships and social acceptance and is an unavoidable prerequisite for independent functioning (28, 65). Thus, autistic individuals are required to exert substantial effort to perform typically “autonomic” aspects of everyday life. Consequently, autistic burnout is characterized by an inability to maintain basic levels of functioning, which is further exacerbated by the intensified presentation of autistic traits (60). Moreover, the inescapable demand to meet the neurotypical threshold for functionality makes recovery from autistic burnout considerably more difficult. Autistic burnout is “pervasive” (59), “chronic” (60), and “recurring” (61); this most closely aligns with a concept known as habitual burnout (68) which describes a state of functional impairment that has become embedded into daily life. Notably, the symptoms of habitual burnout include insomnia, anxiety, behavioral dysregulation, depression, self-doubt, emptiness and isolation (68). The characteristics and consequences of autistic burnout are thus highly specific to its context and present a significant risk for the development of psychopathology (62).

Importantly, autistic burnout is also distinct from clinical depression, despite some superficial similarities. Both MDD and autistic burnout are associated with chronic fatigue, cognitive incapacitation, and suicidal ideation; however, the anhedonia and existential hopelessness that characterizes MDD is not always a defining feature of autistic burnout (60). While depression is described as a lack of *motivation* to participate in life, autistic burnout is experienced as a lack of *capacity* to do so (59). Nevertheless, autistic burnout can lead to the subsequent development of MDD and vice versa. Moreover, the struggle to survive in a constant state of burnout can seem objectively irrational, while the fundamental logistics of living become a physiological impossibility. Thus, autistic burnout represents a distinct, independent and fundamentally different path to suicidal ideation even in the absence of clinical psychopathology (59).

Notably, the interplay between social camouflaging, autistic burnout, and psychopathology is exacerbated in autistic individuals who remain undiagnosed throughout childhood due to an absence of adequate supports and the chronic suppression of autistic traits. Furthermore, undiagnosed autistic individuals lack an explanation for their differences or challenges and tend to internalize these as personal failings (64, 69). A pervasive experience among late-diagnosed autistic individuals is a lifelong sense of “inherent wrongness”, alienation, and a deep sense of shame about who they are (70). This culminates in low self-esteem, a lack of self-worth, and destructive core beliefs that are all risk factors for psychopathology (71). This emphasizes how the lack of awareness and stigma surrounding ASC contributes to autistic burnout and how early diagnosis could play an important role in preventing psychopathology (61). While the link between ASC and psychopathology is well-established, there is a critical gap in the literature on what physiological mechanisms lead to autistic burnout, how they differ from, or contribute to psychopathology, and crucially, how these could be prevented. However, recent progress in molecular neuroscience may reveal an important connection between early life adversity, allostatic load and psychopathology in ASC.

## Early life stress, chronic adolescent stress, and allostatic load in ASC

There is substantial evidence from both epidemiological and preclinical studies that ELS and CAS increase the risk of psychopathology and suicidality in adulthood (72–79). Notably, the established definitions for ELS and CAS closely mirror those emerging for autistic experiences of childhood and adolescence. While it has historically been challenging to derive a universally accepted definition for ELS or CAS, current models agree that stress refers to “*environmental events or chronic conditions that objectively threaten the physical and/or psychological wellbeing of an individual*” (73), undermine a child's “*sense of safety, stability, and bonding*” (80) and for which “*adequate coping resources are unavailable*” (74). Much of the literature focuses on adverse childhood experiences, as ELS during infancy (0–2 yrs old) is thought to disrupt critical periods of post-natal brain development leading to impairments in cognition, language processing, social capacity, and behavioral and emotional regulation (81). However, adolescence is also a critical period for brain development that is highly sensitive to stress. In fact, CAS is known to contribute to the development of depression, anxiety, and other stress-related psychopathologies (73). Researchers have previously distinguished between good, tolerable, and toxic stress; these differentially induce adaptation, resilience or pathology, respectively. Here, the defining feature of toxic stress is “*a lack of internal resources or external support systems, resulting in chronic physiological dysregulation*” (82).

Importantly, the literature suggests that autism is associated with a significantly increased exposure to toxic stress in childhood and adolescence. Children with ASC are statistically more likely to experience early adversity (83), social vulnerability and isolation (84), bullying, discrimination, and ostracism (85), and other forms of victimization (86). Differences with respect to social communication, development and integration can make it difficult for autistic children to feel a sense of “safety and stability” among their peers. Moreover, situations that are “tolerable” for neurotypical children are experienced as “toxic” stressors due to distinct neurophysiological differences in autism. Exposure to every-day sensory stimuli is painful, uncontrollable, and “personally threatening” due to difficulties with sensory processing. Thus, autistic children are pushed beyond their capacity just to achieve a degree of social participation, and even further resources are expended to achieve social acceptance. Personal accounts of social camouflaging describe attempts to manage both the “*physically assaultive*”, “*uncertain, exhausting nature of the social environment*” (87), and the persistent fear of stress, rejection and being misunderstood by peers (88). Social camouflaging has been conceptualized as the discrepancy between the internal experience of autism and the external presentation of autistic traits (22, 56, 58), and is experienced as a need “*to exceed what nature has given*” (27); similarly, autistic burnout has been described as “*having all of your internal resources exhausted beyond measure*” (59). This literature consistently points to a mismatch between external demands and internal capacity, and clearly demonstrates an absence of “adequate coping resources” which is consistent with the accepted definitions for toxic stress.

We propose that social camouflaging could act as a distinct form of CAS in autism. Camouflaging strategies become increasingly complex and intentional from middle childhood, which is a critical period for socialization marked by the development of self-concept and an increasing capacity for self-regulation (89). The self-concept tasks of middle childhood culminate in the ability to internalize expectations for one's own behavior and develop strategies to meet these expectations (89). This enables an autistic child to both identify the need to suppress autistic traits and develop enough self-regulation to implement camouflaging as a replacement for natural behaviors. The extent to which these tasks are completed will vary widely across the autism spectrum depending on differences in both ASC presentations and sociocultural factors that contribute to socialization. Equally, there are significant differences with respect to an individuals' ability and motivation to mask their autistic traits.

This has important implications for the relationship between ASC, chronic stress and psychopathology. Individuals with more extreme sensory sensitivities, greater difficulties with self-regulation and communication, or atypical developmental profiles may be highly vulnerable to increased early life adversity. Yet, this might also facilitate earlier diagnosis, access to accommodations, and a reduced motivation or tendency to engage in chronic camouflaging. Conversely, many individuals with a greater tendency to mask may present with a “subclinical” autism phenotype, but a significant susceptibility to CAS induced by the chronic suppression of autistic traits. It is worth reiterating that social camouflaging refers to internal efforts that are, by definition, removed from external presentations. High levels of masking do not correlate with any particular expression of autism and cannot be inferred based on the nature or degree of observable autistic traits. Rather, this emphasizes that different external autism presentations are not always reflective of the profound physiological and psychological dysregulation experienced internally. Despite the heterogeneity of ASC, an underlying susceptibility to stress-related pathologies could be a common mechanism responsible for negative clinical outcomes in this context.

The resonance between internal experiences of autism and established models for toxic stress provides a point of convergence between psychosocial and molecular research into ASC. From a molecular standpoint, ELS and CAS are thought to contribute to allostatic load, which describes the pathophysiological consequences of chronic stress on the metabolic, endocrine and immune systems required for homeostasis (40). Allostatic load provides an integrative model to quantify the cumulative impact of biological, psychological and social factors that contribute to pathology (40). In this context, social camouflaging could serve as a unique form of toxic stress that increases allostatic load in autism. Autistic burnout may represent a distinct pathophysiological state resulting from allostatic overload, culminating in neurological and psychological dysregulation that leads to psychopathology. Previous work has described how allostatic load may interact with behavioral, psychosocial and physiological aspects of ASC (37–39, 90); however, this model remains poorly characterized from a molecular perspective. Notably, the molecular mechanisms that mediate the association between ELS, CAS and psychopathology are also involved in ASC etiology. This could signify an underlying molecular susceptibility to ELS that may yield mechanistic insight into the relationship between autistic burnout, psychopathology and suicidality.

## Mitochondrial allostatic load and convergent molecular mechanisms

The concept of allostatic load is well-established (91–93), but the specific molecular mechanisms that mediate pathological stress responses remain poorly characterized (94). The HPA axis has traditionally been the focus of stress pathophysiology research in ASC; however, the evidence for HPA axis dysregulation in autism is inconsistent (95). Recently, mitochondrial metabolism has been recognized as a central regulator of the major neuroendocrine and neuroimmune systems involved in allostasis (40). Emerging evidence highlights the particular relevance of mitochondrial allostatic load (MAL), which describes adaptive changes to mitochondrial morphology, dynamics, and function in response to chronic stress (93). Notably, mitochondrial dysfunction is a biological signature that is not only consistently observed in idiopathic ASC, but also independently implicated in ELS and psychopathology. Thus, MAL could serve as a central molecular mechanism involved in the development of psychological disorders in autistic individuals.

### Mitochondrial allostatic load

A preliminary but growing body of evidence suggests that mitochondrial dysfunction is involved in the relationship between ELS and psychopathology (96–98). Clinical studies have shown that ELS increases mitochondrial respiratory activity (99, 100), mitochondrial DNA (mtDNA) copy number (101, 102), and circulating cell-free mtDNA (103, 104), all of which indicate a dysregulation of mitochondrial function. Epidemiological studies also demonstrate that ELS disrupts typical indicators of redox homeostasis, including reactive oxygen species (ROS), antioxidant enzymes like superoxide dismutase (SOD) and glutathione peroxidase, and total reactive antioxidant potential (105, 106). This is reflected in animal models for ELS showing decreased SOD and increased protein carbonylation, ROS and glutathione (107, 108). Preclinical studies further demonstrated that ELS impairs mitochondrial electron transport chain (ETC) activity in the hypothalamus and dysregulates mitochondrial fission and antioxidant defense genes in the hippocampus, leading to cognitive impairments (109). Moreover, a recent systematic review presented consistent evidence for reduced mitochondrial energy production capacity and ETC complex activity, altered mitochondrial morphology and changes in mtDNA copy number in animal models for chronic stress (110). Notably, the duration and type of stress, as well as underlying genetic differences significantly affect how stress alters mitochondrial function (110). Conversely, experimentally disrupting mitochondrial function alters the physiological and behavioral consequences of psychological stress (40).

Similarly, mitochondrial dysfunction has been widely implicated in the pathogenesis of MDD; this has recently been comprehensively reviewed (93, 111–117). Mood disorders are frequently reported in patients with mtDNA mutations and up to half of patients with primary mitochondrial diseases present with MDD. Conversely, depression is associated with elevated mtDNA deletions, aberrant mtDNA copy number and oxidative mtDNA damage (113, 118, 119). Independent studies have reported increased oxidative stress and altered ETC activity (120) and decreased levels of antioxidants and antioxidant enzymes (121) in the brain tissue of patients with depression. Of note, Karabatsiakis et al. (122) reported significantly impaired mitochondrial metabolism in individuals with MDD, which correlated significantly with the severity of depressive symptoms. Pre-clinical studies also demonstrate an increase in ROS and a decrease in ATP and glutathione in models for depression (113). Importantly, many pre-clinical models for MDD are induced by ELS, and consistently implicate mitochondrial mechanisms in the subsequent development of depressive behaviors (116). For example, *in vivo* models for MDD have shown that mitochondrial ETC complex activity is disrupted in brain tissue following maternal deprivation, social isolation, restraint stress, mild stress, and unpredictable stress (123–128). Moreover, several transcriptomic studies have reported significant dysregulation of mitochondrial genes involved in lipid metabolism, oxidative phosphorylation (OXPHOS) and glucose homeostasis in preclinical models for chronic and acute stress (114, 129–131). Thus, both epidemiological studies and animal models support a role for mitochondrial dysfunction in the relationship between ELS and MDD. Indeed, a recent review describes the central role of MAL in the symptoms of MDD and emphasizes the utility of targeting mitochondrial function to develop novel pharmacological treatments (93).

Mitochondrial dysfunction is also a widely-studied molecular signature associated with ASC; thus, the interplay between ELS and MAL is particularly relevant in this context. A seminal 1998 review of ASC clinical data demonstrated decreased cortical glucose uptake and ATP synthesis, as well as plasma lactic acidosis, carnitine deficiency and elevated urinary Krebs cycle metabolites (132). Subsequently, multiple lines of evidence have emerged to support a role for mitochondrial dysfunction in ASC and these are well-described in recent reviews (133–139). Noteworthy, clinical studies have reported altered mtDNA copy numbers and deletions (136, 140–143), mtDNA mutations (144, 145), mitochondrial ETC deficits (140, 142, 146) and altered plasma levels of lactate, pyruvate, alanine, creatine kinase, glutathione-S-transferase and caspase 7 (144, 147). Recent studies in ASC-derived lymphoblastoid cell lines (LCLs) showed significantly increased mitochondrial respiration and mitochondrial membrane potential as well as elevated activities of ETC complexes (148), while disruptions to mitochondrial bioenergetics, dynamics and morphology were observed in ASC-derived fibroblasts (149, 150). This is supported by independent transcriptomic studies that have reported reduced expression of mitochondrial respiration genes in ASC brain tissue (151–154). In fact, ASC transcriptomic, proteomic and DNA methylation functional enrichment signatures consistently converge on mitochondrial OXPHOS (155). Furthermore, increased oxidative stress is well-documented in ASC, with recent reviews discussing the cumulative evidence for altered glutathione metabolism, lipid peroxidation, protein oxidation, DNA oxidation and antioxidant enzyme activity in ASC (156–161). Thus, there is substantial evidence for altered mitochondrial metabolism in ASC, which could serve as a molecular susceptibility to the pathological consequences of MAL.

Notably, the relationship between MAL and ELS has only recently emerged, and the limitations of the current state of knowledge have been comprehensively evaluated (97). The field is still developing quantitative cell-specific techniques and more integrative computational approaches to better characterize the complex and dynamic role of mitochondria in psychopathology and disease. Only recently have clinical studies explored indices of MAL, and these studies may be confounded by genetic and environmental factors that affect mitochondrial function (96). Nevertheless, the evolving field of mitochondrial psychobiology (97) represents a novel avenue for future research to explore a role for MAL in ASC clinical outcomes. Moreover, mitochondria are mechanistically implicated as essential modulators of both the neuroendocrine and neuroimmune components of allostasis (40, 96), each of which are thought to play a role in ASC.

### MAL and the HPA axis in stress-related psychopathology

The HPA axis is the major neuroendocrine system involved in initiating the stress response and coordinating the molecular components of allostatic load (73). Children are particularly vulnerable to HPA disruption during early childhood and adolescence, which are periods marked by higher HPA axis plasticity. Thus, HPA signaling is a key mediator of pathological responses to ELS and CAS (73, 81). A recent systematic review has demonstrated an established role for HPA axis dysregulation in mood disorders, suicidal behavior, and the relationship between ELS and major depression (162). Transcriptomic studies in brain tissue suggest that HPA axis signaling is involved in the development of MDD following ELS (72, 163). Furthermore, disruptions to cortisol and catecholamine signaling have been reported in individuals with a history of ELS who develop PTSD, depression and suicidality (164). Recent work in animal models has also shown distinct perturbations to HPA axis signaling, stress reactivity, and cognitive and emotional functioning in response to both ELS and CAS (165).

**Figure 1:**
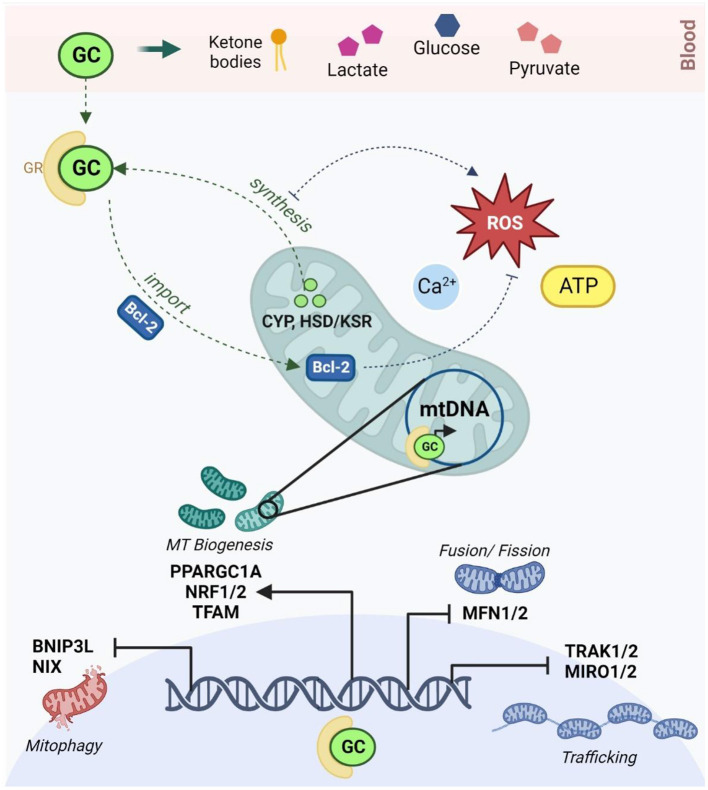
The reciprocal relationship between glucocorticoid signaling and mitochondrial allostatic load (created with BioRender.com). HPA activation in response to chronic stress leads to the release of GCs which diffuse through the blood-brain barrier to activate GRs in neurons. GCs alter circulating levels of key energetic substrates including ketone bodies, glucose, lactate and pyruvate, which modulates mtFAO, glycolysis and OXPHOS. Activated GRs act *via* Bcl-2 and other non-genomic mechanisms to modulate membrane potential, calcium buffering, ATP production and redox homeostasis in the mitochondria. Activated GRs also bind to glucocorticoid response elements in both nuclear and mitochondrial DNA to moderate the transcription of key genes involved in the regulation of mitochondrial trafficking (TRAK1/2, MIRO1/2), morphology (MFN1/2), biogenesis (PPARGC1A, TFAM, NRF1/2) and mitophagy (BNIP3, NIX). Conversely, mitochondria are responsible for the synthesis of GCs *via* the CYP540 and HSD/KSR enzymes. GC synthesis increases mitochondrial ROS, and conversely, oxidative stress is known to inhibit steroidogenesis. HPA, hypothalamic-pituitary-adrenal; GCs, glucocorticoids; GRs, glucocorticoid receptors; mtFAO, mitochondrial fatty acid oxidation; OXPHOS, oxidative phosphorylation; ROS, reactive oxygen species; Bcl-2, B-cell lymphoma 2; MFN1/2, mitofusin 1 and 2; TRAK1/2, Trafficking Kinesin Protein 1/2; MIRO ½, Mitochondrial Rho GTPase; BNIP3, Bcl2 Interacting Protein 3; NIX, BNIP3-like; PPARGC1A, peroxisome proliferator-activated receptor gamma coactivator-1 alpha; TFAM, mitochondrial transcription factor A; NRF1/2, NRF1/2 nuclear respiratory factors 1 and 2; CYP, cytochrome P450; HSD/KSR, hydroxysteroid dehydrogenase/ketosteroid reductase.

Importantly, a bidirectional relationship is emerging between the HPA axis and mitochondrial metabolism (Figure 1). Stress-responsive glucocorticoids (GCs) like cortisol and corticosterone (166) are released in response to HPA axis activation and act on GC receptors (GRs) to regulate genes involved in neurogenesis, neuroplasticity, and neurotransmission (167). Mitochondria are directly responsible for the synthesis of GCs *via* cytochrome P450 enzymes that are coupled to mitochondrial redox state (168). Mitochondrial metabolism not only regulates, but is also modulated by, GC signaling (40, 102); this relationship is mediated by both genomic and non-genomic mechanisms that have been comprehensively reviewed (169–176). At physiological levels, short-term GC exposure improves mitochondrial oxidation, membrane potential and calcium buffering, while chronic GC treatment reduces the activity of specific mitochondrial ETC complexes, increases mitochondrial ROS production, and upregulates mitochondrial fragmentation (175, 177–180). Moreover, GCs affect the availability and turnover of key energetic substrates in the brain, including pyruvate, glucose, lactate and ketone bodies which regulate mitochondrial OXPHOS, glycolysis and fatty acid oxidation, respectively (169). GCs also modulate mitochondrial dynamics by regulating the expression of relevant genes *via* GC response elements (GREs) in both nuclear and mtDNA (172–174). GCs have been shown to downregulate the transcription of the genes involved in mitochondrial fusion, trafficking and clearance (170, 180) and increase the expression of several central transcriptional regulators of mitochondrial biogenesis (102, 174, 181). Notably, animal models have shown that both chronic stress and corticosterone treatment increase mtDNA copy number (119), and conversely, that mtDNA genetic variants can alter corticosterone production and HPA signaling (182, 183). Additionally, epidemiological studies have shown that mtDNA copy number is elevated in people with a history of ELS (184) and in major depression (119), while recent work demonstrated that the relationship between ELS and mtDNA copy number was mediated by differential methylation of the GR (184). Thus, both mitochondrial function and GC signaling are independently implicated in the response to ELS and MDD and recent findings suggest that these mechanisms are interdependent.

Interestingly, this may reveal a novel molecular susceptibility to ELS that has been overlooked in the context of ASC. Although much research has investigated a role for HPA signaling in the relationship between autism and psychopathology, this has yielded inconsistent results. There is a large body of evidence suggesting global disruptions to cortisol signaling in autism (95, 185–193) and independent studies have reported HPA hyperactivation (185, 188, 194, 195) and increased stress reactivity (186, 196) in ASC. A relationship between elevated evening cortisol and psychopathology in ASC has been proposed (194); altered cortisol responses were associated with GAD and social anxiety in boys with ASC (197, 198), while the cortisol awakening response was reported to correlate with depression and suicidal ideation in autistic girls (199). Moreover, targeted molecular studies have found disruptions to GR expression in ASC brain tissue (200), while genetic polymorphisms in the GR chaperone were associated with serum cortisol levels (201) and moderated the relationship between autistic traits and social anxiety (202). However, several reviews have summarized the conflicting evidence for HPA dysregulation, and its association with psychopathology, in ASC (46, 95, 185–187, 203). These authors emphasize that current findings lack intra- and inter-individual reproducibility, while numerous studies report no significant differences in cortisol levels, rhythms, variability or responsivity in ASC. Of note, Muscatello et al. (46) found no relationship between HPA activation, depression and anxiety in autistic children; instead reporting a complex multi-systemic relationship between HPA signaling and psychopathology. Yet, many studies assess HPA function using end-point measurements of salivary cortisol, which cannot always reflect complex interactions between biological signaling processes and genetic architecture with sufficient resolution. The existing literature suggests some role for the HPA axis in ASC etiology; however, the relationship between HPA axis signaling and psychopathology in autism is multifaceted and likely mediated by pleiotropic biological factors that contribute to an increased susceptibility to HPA axis dysregulation (46). In this context, the emerging relationship between mitochondrial metabolism and HPA signaling highlights mitochondrial dysfunction as a common biological signature that could act as a novel susceptibility to stress-related psychopathology in ASC.

### The neuroimmune system: A link between MAL and the HPA stress response

The neuroimmune system is the second established component of allostatic load that works in concert with HPA neuroendocrine signaling to modulate the stress response. Neuroimmune dysregulation is well-documented in clinical studies of neuropathology and is observed in response to ELS (74, 204, 205), and neurodevelopmental (206) and psychological (207–209) disorders. In fact, Bottaccioli et al. (210) proposed that the “neuroendocrine-immune” network is a central molecular mediator between psychosocial stress and the psyche. Accordingly, preclinical models have demonstrated that chronic stress consistently induces microglial activation and proinflammatory cytokine signaling in key brain regions involved in psychiatric disorders (205). Moreover, recent animal studies showed that ELS-induced depressive behaviors were associated with neuroinflammation (108, 211, 212) and aberrant microglial activity (213), while CAS was also shown to increase hippocampal immune reactivity (214). Furthermore, disruptions to microglial phagocytosis impaired neuronal development, cognitive performance, memory, reward processing, and processing of social stimuli (215). Notably, there is also extensive evidence for neuroinflammation in ASC (160, 216–221). Inflammatory cytokines are reported to be elevated in ASC blood and post-mortem brain tissue, while immunohistochemistry, positron emission tomography and morphological data demonstrates glial over-proliferation and over-activation in ASC (222–227). Transcriptomic studies have shown that genes involved in inflammation, astrocyte function and microglial activation were significantly dysregulated in ASC brain tissue (228), including the glial-specific marker GFAP which was overexpressed at both the mRNA and protein levels (229). Post-mortem studies also found a significant upregulation of microglial-specific genes (226), increased microglial density (224, 230) and excessive microglial activation (225, 231) in ASC brain tissue, while two recent systematic reviews report consistent evidence for disrupted microglial morphology, organization and activation in autism (220, 232). Although the precise nature of neuroimmune dysregulation is context-specific and pleiotropic in both ASC and ELS (204, 233), these data reveal an interesting mechanistic overlap between ASC neurology and stress-related psychopathology.

The neuroimmune system also acts as an intermediary between MAL and the HPA axis on a molecular level. Firstly, mitochondrial metabolism is a central modulator of glial cell phenotypes and neuroinflammatory state (40). Glial functions are controlled by tightly regulated interactions between microglia, astrocytes, oligodendrocytes and neurons, which are closely coupled to glial metabolism (234). Glial phenotypes are dynamic, shift in response to different stimuli and can be either neuroprotective or neurotoxic (235). The transition to an inflammatory phenotype is known as reactive gliosis, which is characterized by transcriptional, biochemical, metabolic and morphological re-modeling (236). This has distinct implications for the metabolism, physiology and function of different glial cell types (237–242). While microglia, astrocytes and oligodendrocytes are each implicated in specific aspects of ASC neurology (216, 220, 243–246), microglial phenotypes are particularly relevant in the context of neurodevelopment. Microglia are the only glial cells present in the early embryonic brain (247) that play an essential role in the regulation of neurogenesis, synaptic activity and axonal myelination (242, 248–250), all of which are dysregulated in autism (216, 251, 252). Moreover, a cohesive body of work from animal models suggests that microglia are centrally involved in the neurological and behavioral consequences of ELS (253).

**Figure 2:**
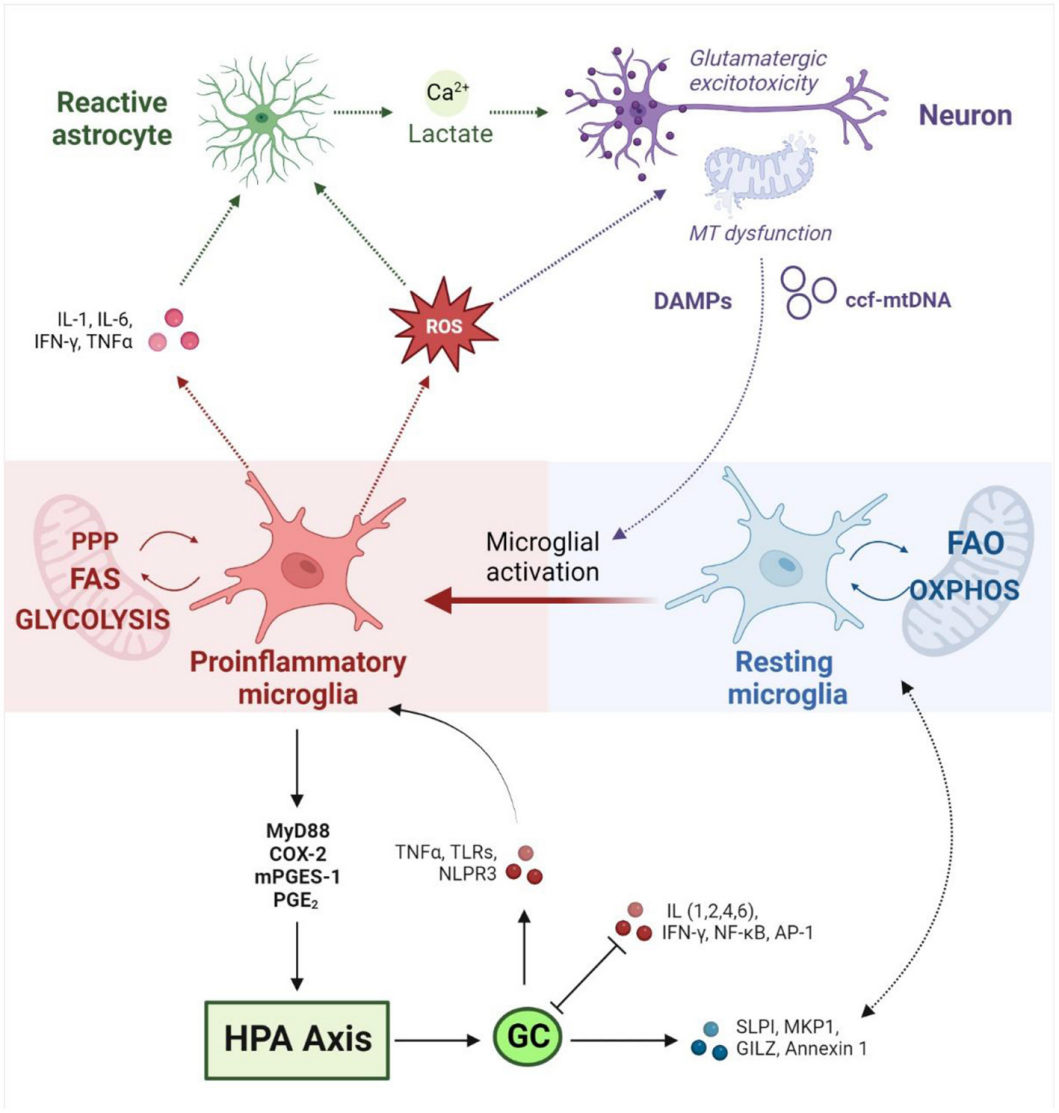
Neuroimmune responses at the interface between mitochondrial metabolism and the HPA axis (created with BioRender.com). Neuroimmune homeostasis is maintained by a tightly regulated interplay between dynamic glial phenotypes. Microglial activation toward a pro-inflammatory state is driven by intrinsic metabolic reprogramming away from OXPHOS and FAO toward glycolysis, FAS and the PPP. Conversely, microglial metabolic profile directly influences inflammatory state. Activated microglia release pro-inflammatory cytokines and ROS, which in turn activates neighboring astrocytes and induces mitochondrial dysfunction in neurons. Reactive astrocytes release lactate and calcium, which upregulates glutamatergic neurotransmission and culminates in an excitotoxic loop that drives further inflammation and oxidative stress. Damaged mitochondria in neurons induce the release of DAMPs and ccf-mtDNA which induce pro-inflammatory signaling in microglia. Inflammatory stimuli drive hypothalamic CRH production *via* MyD88, COX-2, mPEGS-1 and PGE2. HPA activation leads to the release of GCs which mediate inflammatory signaling on several fronts. GCs promote anti-inflammatory responses *via* SLP1, MKP-1, GILZ and Annexin 1, while downregulating pro-inflammatory signaling by IL-1,2,4 and 6, NF-κB, IFN-γ, and AP1. GCs have also been shown to upregulate inflammatory pathways *via* TLRs, TNF-α and the NLRP3 inflammasome. Conversely, pro-inflammatory cytokines interfere with GC signaling by disrupting GR expression, phosphorylation and nuclear translocation. MT, mitochondrial; HPA, hypothalamic-pituitary-adrenal; OXPHOS, oxidative phosphorylation; FAO, fatty acid oxidation; FAS, fatty acid synthesis; PPP, pentose phosphate pathway; ROS, reactive oxygen species; DAMPs, Damage Associated Molecular Patterns; ccf-mtDNA, circulating cell-free mitochondrial DNA; GCs, glucocorticoids; CRH, corticotrophin-releasing hormone; MyD88, Myeloid differentiation primary response 88; COX-2, Prostaglandin-endoperoxide synthase 2; mPEGS-1, microsomal prostaglandin E synthase; PGE2, prostaglandin E2; SLP1, secretory leukocyte protease inhibitor; MKP-1, mitogen-activated protein kinase phosphatase-1; GILZ, glucocorticoid-induced leucine zipper protein; IL, Interleukin; NF-κB, Nuclear factor kappa-light-chain-enhancer of activated B cells; IFN-γ, Interferon gamma; AP-1, activator protein 1; TLRs, toll-like receptors; TNF-α, tumor necrosis factor α; NLRP3, NLR family pyrin domain containing 3.

Importantly, mitochondrial dysfunction can induce microglial activation *via* both extrinsic and intrinsic mechanisms (Figure 2) (235, 254). Mitochondrial dysfunction in surrounding brain tissue leads to the release of damage-associated molecular patterns (DAMPs) that bind to microglial receptors and induce pro-inflammatory pathways (137). Moreover, microglial activation is marked by intrinsic metabolic reprogramming, and different microglial phenotypes are associated with distinct metabolic pathways (249). While resting microglia rely primarily on OXPHOS (255), the shift toward the inflammatory M1 phenotype is driven by a decrease in mitochondrial respiration and an upregulation of glycolysis. The latter enhances flux through the pentose phosphate pathway and increases lactate production to meet increased cellular energy demands (235). Recent work has demonstrated that mitochondrial metabolism directly regulates microglial activation (256, 257) and that disrupting mitochondrial function has profound implications for microglial inflammatory state (248, 249, 257, 258). *In vitro* studies have shown that inhibiting mitochondrial ETC activity (259) or increasing mitochondrial fragmentation (249) induces microglial activation and pro-inflammatory cytokine signaling; this could be mitigated by targeting mitochondrial fission (260), membrane potential, or ROS (249). Concurrently, mitochondrial stress impairs the transition to the anti-inflammatory M2 phenotype (261), which exacerbates neuroinflammation and oxidative stress (262). Conversely, different cytokine stimuli are known to alter microglial metabolic state. Inflammatory stimuli were found to induce a metabolic switch from OXPHOS to glycolysis in microglia *in vitro* (263), marked by increased lactate production and decreased OXPHOS, oxygen consumption and ATP production (264, 265). This metabolic shift correlated with increased production of proinflammatory cytokines (266) and nitric oxide (263), demonstrating a proinflammatory feedback loop driven by metabolic reprogramming. Ultimately, this highlights an intrinsic coupling between the neuroimmune and mitochondrial mechanisms implicated in allostatic load.

Stress-responsive neuroendocrine mediators are also known to modulate immune responses (40), and the bidirectional relationship between inflammatory- and GC- signaling has been well-documented in the context of psychopathology (106, 209, 267–270). GRs mediate anti-inflammatory effects *via* the transcriptional transrepression of pro-inflammatory genes such as nuclear factor kappa-light-chain-enhancer of activated B cells (NF-κB) and the activation of anti-inflammatory genes like annexin 1 (171, 268, 270). GRs also regulate key pathways involved in cell proliferation and differentiation to inhibit immune cell activation and inflammatory cytokine production. On the other hand, GCs have been shown to upregulate pro-inflammatory pathways *via* toll-like receptors, tumor necrosis factor α (TNF-α) or the NLRP3 inflammasome (209). Conversely, inflammatory cytokines modulate GC signaling by disrupting GR expression, phosphorylation and nuclear translocation (271). Cytokine-stimulated immune cells can also promote HPA axis activation and GC signaling by upregulating the production of corticotrophin-releasing hormone (CRH) in the hypothalamus (181). Of particular relevance, animal models have demonstrated an antagonistic relationship between HPA activity and interleukin 6 (IL-6), which is one of the most consistently upregulated inflammatory markers associated with ELS (204), MDD (209) and ASC (272) that has also been widely implicated in psychological disorders (273). Markedly, recent preclinical studies suggest that neuroimmune-HPA interactions facilitate the neuroinflammatory response to stress. ELS was shown to increase GR promoter methylation and decrease GR expression, which was associated with upregulated proinflammatory NF-κB signaling (274). Similarly, CAS led to a significant transcriptional dysregulation of GR signaling, increased NF-κB signaling and excessive microglial activation in female rats (275).

Given that GR signaling regulates mitochondrial function, and that mitochondria are central to immune modulation, it has been proposed that HPA-immune interactions could be mediated by mitochondrial mechanisms (40, 171, 276). Mitochondria co-ordinate several of the inflammatory pathways at the interface between HPA and immune signaling, including NF-κB, IL-6, and inflammasome signaling (99). The GC-dependent NF-κB and MAPK signaling pathways are also intrinsically coupled to central regulators of mitochondrial biogenesis and homeostasis, including PPARGC1A (277). Moreover, GCs have been shown to upregulate the secretion of cell-free mtDNA *in vitro*, which in turn, serves as a potent activator of inflammatory signaling (103). Congruent with this, recent data demonstrate that ELS concurrently impairs mitochondrial function and increases neuroinflammation in animal models (278). Additionally, clinical studies report a significant correlation between mitochondrial respiration, oxidative stress and the production of pro-inflammatory cytokines, including IL-6, following ELS (99). Collectively, this reveals a tightly coupled interplay between mitochondrial metabolism, neuroimmune and neuroendocrine signaling that underlies the established relationship between ELS, MAL and psychopathology. This discussion has highlighted evidence implicating each of these molecular stress signatures in ASC (Table 1), emphasizing how mitochondrial dysfunction plays a central role in modulating the neuroimmune and neuroendocrine stress responses that contribute to allostatic load. Undoubtedly, further work is required to fully elucidate the molecular relationships between the HPA axis, MAL and neuroinflammation, and to better characterize how these mechanisms contribute to clinical aspects of ASC. Nevertheless, this model points to a molecular signature that could serve as an underlying susceptibility to ELS, and may contribute to the high rates of psychopathology and suicidality in ASC.

**Table 1:** Molecular components of allostatic load implicated in ASC.

**Component of allostatic load**	**Evidence of dysregulation in ASC**	**Original reports**	**Meta analyses and reviews**
Mitochondrial	Transcriptomic, proteomic and epigenomic mitochondrial signatures Mitochondrial DNA copy number, deletions and mutations Altered electron transport chain function, mitochondrial membrane potential, bioenergetics, dynamics and morphology Increased lactic acidosis and Krebs cycle metabolites, decreased carnitine and altered pyruvate, alanine, creatine kinase, glutathione-S-transferase and caspase 7 in plasma Decreased glucose uptake, ATP synthesis & transcription of OXPHOS genes in brain tissue Dysregulated glutathione metabolism, lipid peroxidation, protein oxidation, DNA oxidation and antioxidant enzyme activity	(136, 140, 142–144, 146–153)	(132–135, 137–139, 141, 145, 154–161)
Neuroendocrine	Global disruptions to cortisol signaling Hypothalamic–pituitary–adrenal (HPA) hyperactivation and increased stress reactivity Disrupted glucocorticoid receptor (GR) expression in brain tissue Mutations in GR cochaperone associated with serum cortisol and social anxiety Cortisol awakening response, evening cortisol and signaling between the HPA and autonomic nervous system correlated with psychopathology, anxiety, depression and suicidal ideation	(46, 185, 188, 193–196, 199–202)	(46, 95, 186, 187, 191, 192)
Neuroimmune	Elevated inflammatory cytokines in blood and brain tissue Reactive gliosis, increased microglial density and activation and disrupted microglial morphology and organization Dysregulated transcription of genes involved in inflammation, astrocyte function and microglial activation and significant upregulation of microglial-specific genes	(222–230)	(160, 216–221, 232)

### From MAL to neurophysiology, neurochemistry, and behavior: A mechanistic perspective

**Figure 3:**
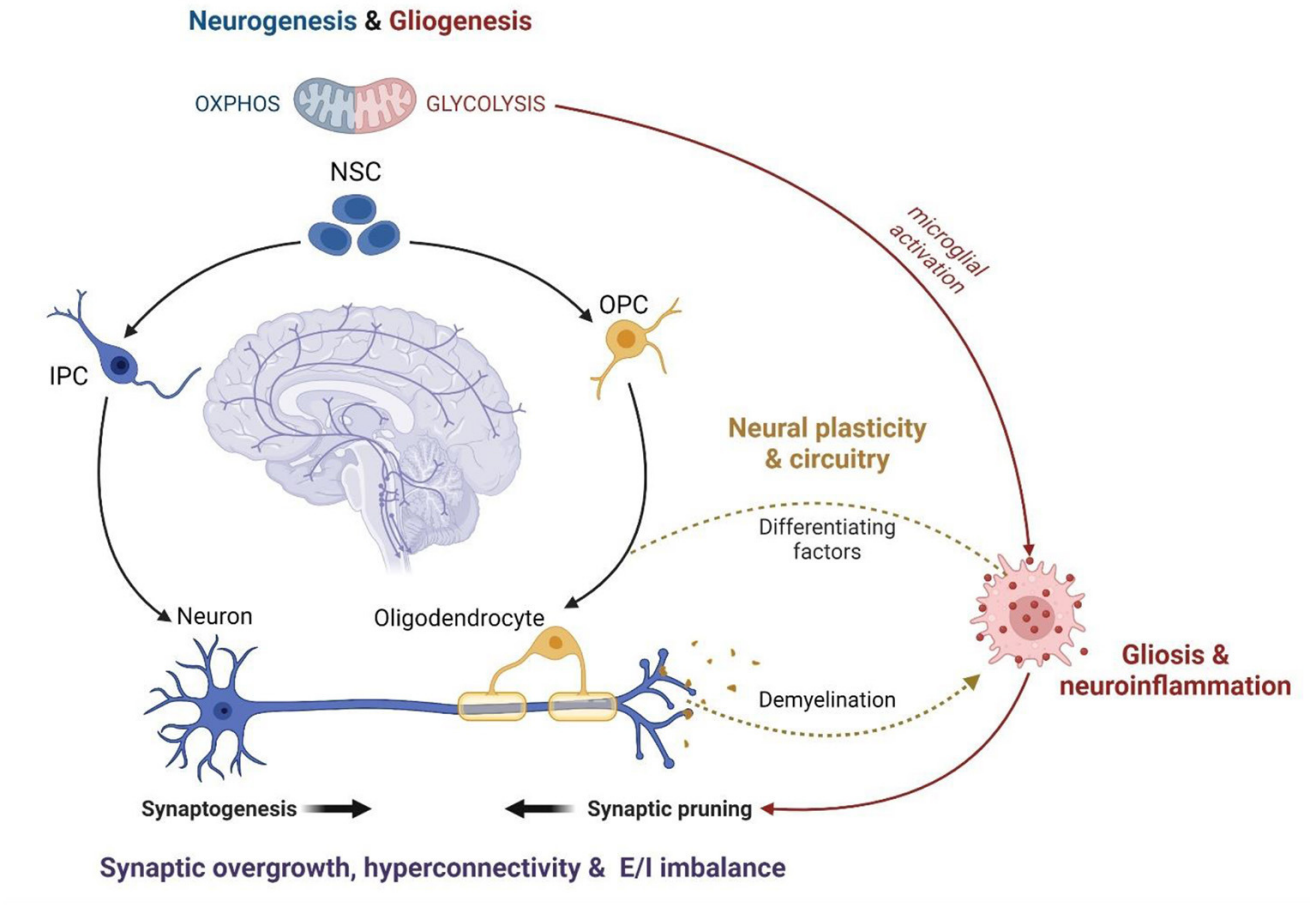
The mitochondrial modulation of ASC neurophysiology (created with BioRender.com). Mitochondrial metabolism functions as a central regulator of NSC self-renewal and differentiation. Neuronal differentiation is driven by a significant increase in OXPHOS while stem cell fate is shifted toward gliogenesis under oxidative and metabolic stress. Thus, mitochondrial dysfunction disrupts neurodevelopment and contributes to glial over-proliferation and subsequent neuroinflammation. The remodeling of mitochondrial metabolism also facilitates the maturation of myelinating oligodendrocytes and modulates the activation of microglia during neurodevelopment. Microglial activation is essential for post-natal synaptic pruning, which is dysregulated in ASC leading to synaptic overgrowth, cortical hyperconnectivity, and an imbalance between excitatory and inhibitory synapses. Anti-inflammatory microglia release growth factors that promote oligodendrocyte maturation while pro-inflammatory microglia are responsible for demyelination *via* phagocytosis. The coupling between mitochondrial fatty acid metabolism and glial phenotypes underlies the oligodendrocyte-microglial interactions that modulate myelin synthesis and turnover, which is crucial for the formation and plasticity of neural networks. Therefore, mitochondrial dysfunction is a common mechanism implicated in several features of ASC neurodevelopment, physiology and function. NSC, Neuronal Stem Cell; IPC, Intermediate Progenitor Cell; OPC, Oligodendrocyte Progenitor Cell; OXPHOS, oxidative phosphorylation; E/I imbalance, excitatory/inhibitory imbalance.

A role for MAL in the relationship between autism and psychopathology is supported by mounting evidence that mitochondria are centrally involved in neurodevelopment, neurophysiology and neurochemistry. In fact, mitochondria are known to modulate distinct neurological processes that are disrupted in response to ELS and involved in the etiology of ASC. ELS alters the proliferation, differentiation and survival of neuronal stem cells (NSCs), culminating in disruptions to synaptogenesis, synaptic pruning, and neurotransmission (72, 164). Extensive synaptic remodeling also occurs during adolescence, and CAS is known to disrupt synaptic plasticity and myelination with long-term implications for psychology, cognition and behavior (73, 279). These neurophysiological differences have largely been attributed to signaling between the HPA axis, GCs and neurogenic factors (e.g., Brain-derived neurotrophic factor and Wnt) (233, 234). However, just as the concept of MAL is becoming increasingly established in stress research, mitochondria are also emerging as central regulators of neurodevelopment and physiology (Figure 3).

The balance between NSC self-renewal and neuro-, oligo- and astrogenesis is closely tied to metabolic state. Neurogenesis requires a metabolic shift from glycolysis to OXPHOS while oxidative and metabolic stress drive stem cell fate toward gliogenesis (280–283). Moreover, distinct alterations to mitochondrial morphology and function are observed during the maturation of myelinating oligodendrocytes (284, 285). Metabolic state is coupled to, and regulated by, mTOR signaling, and each are essential for synaptic signaling, function and plasticity (286–295). In fact, preclinical data suggest that ELS impairs neuronal metabolism and synaptic plasticity *via* mTOR-dependent mechanisms (296, 297). The interplay between mitochondrial metabolism and microglial activation is also essential to regulate neuronal apoptosis, synaptic pruning and myelination during neurodevelopment (238, 242, 298). Synaptogenesis is tightly regulated to shape neural circuitry and connectivity (243, 299) while myelin processing is vital for the activity, synchronization and dynamics of neural networks (300). Notably, disruptions to mTOR signaling (289, 301, 302), synaptic pruning (232, 303) and myelination (245) are hallmarks of ASC that contribute to cortical hyperconnectivity and the resultant behavioral phenotypes (250, 299). Thus, the mitochondrial modulation of neuronal development, plasticity and circuitry converges on shared neurophysiological mechanisms in ELS and ASC.

In addition, disruptions to neurotransmitter signaling and neuroendocrinology are implicated in both ELS and ASC. Preclinical studies have shown that chronic stress disrupts glutamate- (304–307), serotonin- (308) and dopamine-(309–312) signaling, leading to increased anxiety-like behaviors and impairments in social recognition, social interest and cognitive flexibility. These data are supported by human cohort studies showing that ELS disrupts dopaminergic responses to psychosocial stress (313, 314), and alters glutamate/glutamine cycling (315) and serotonin transporter binding (316) in patients with MDD. In fact, genetic mutations in the serotonin receptor have been shown to mediate the effect of ELS on the subsequent rates of depression and suicidality (317). CAS also alters neuroendocrinology on several fronts, by inducing glutamatergic excitotoxicity and decreasing levels of serotonin, dopamine and norepinephrine (73). Notably, an unbalanced excitatory-to-inhibitory synaptic signaling ratio (318–320) and disruptions to glutamatergic (246, 320–325), serotonergic (326–330) and dopaminergic (319, 331, 332) neurotransmission are well-established aspects of ASC neurochemistry that are associated with distinct aspects of ASC etiology. Glutamatergic excitotoxicity is thought to contribute to anxiety, perseveration, motor stereotypies, and differences in language, social memory and cognition (325). Serotonin signaling is linked to altered social cognition, facial recognition, emotion processing and communication (329) while dopamine signaling is associated with disruptions to sleep, mood and attention in ASC (319, 331, 332).

This underlying neuroendocrinological dysregulation is not only associated with core facets of ASC etiology, but also has direct functional implications for stress-related psychopathology. A disruption of glutamatergic and GABAergic signaling contributes to the etiology of anxiety disorders (333–336), PTSD (337), eating disorders (338–340), OCD (341) and substance use disorders (SUDs) (342, 343). The serotonergic, dopaminergic and noradrenergic systems are implicated in MDD, anxiety and SUDs (344–346) while serotonin is also associated with mood disorders, stress, aggression and anti-social conduct (347–349), and dopamine is thought to play a role in eating disorders (350, 351), executive dysfunction and behavioral inhibition (352, 353). Moreover, well-documented interactions between neurotransmitter, neuroimmune and neuroendocrine pathways play a central role in modulating suicide risk after ELS exposure (354). Glutamate is involved in both driving HPA responses and limiting HPA overactivation (355–357), and conversely, GC signaling is known to modulate glutamatergic synapse plasticity and excitability (358). Serotonergic neurotransmission excites CRH-producing neurons in the amygdala (209) and acts as an important regulator of GR signaling in the pre-frontal cortex after exposure to acute stress (359). On the other hand, animal models show that corticosteroids decrease serotonin receptor binding densities (360, 361) and this relationship is implicated as a mediator of suicidality following ELS (317). Finally, GCs also activate the mesolimbic dopamine pathway (362, 363) by upregulating the rate-limiting step of dopamine synthesis, downregulating dopamine degradation, clearance and synaptic uptake, or by acting directly on GRs in dopamine-receptive neurons (364).

**Figure 4:**
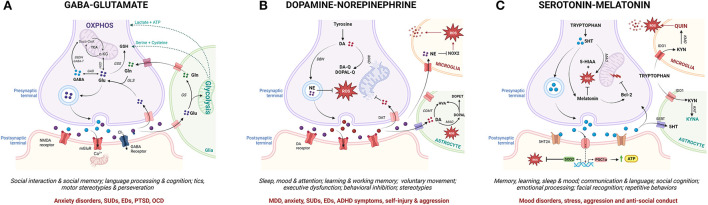
From mitochondrial allostatic load to neurochemistry, behavior and psychopathology (created with BioRender.com). Mitochondrial metabolism drives the synthesis and cycling of key neurotransmitters involved in ASC-associated behavior and psychopathology. While specific neuronal sub-types express subsets of the enzymes involved in the metabolism of each neurotransmitter, each neurotransmitter pathway is depicted in one diagram for the sake of simplicity. **(A)** Glutamine (Gln) and Glutamate (Glu) are synthesized in the mitochondria of glutamatergic neurons and glial cells. Glutamate can be directly integrated into the TCA cycle *via* GDH, converted into GABA *via* GAD or shunted into the transulfuration pathway *via* GSS. After glutamate is released from presynaptic neurons, a small percentage is taken up by post-synaptic glutamate receptors which mediate post-synaptic impulses *via* calcium-dependent signaling cascades. Chronic glutamatergic activation leads to an influx of calcium and mitochondrial dysfunction in post-synaptic neurons. The majority of presynaptically-released glutamate diffuses out of the synaptic cleft and is taken up by glial cells where it is either integrated into the TCA cycle or converted to glutamine by GS. Glial glycolysis also supplies neurons with key substrates for the synthesis of the antioxidant GSH. **(B)** In dopaminergic neurons, DA is either deaminated by MAO or auto-oxidized by mitochondrial-derived ROS to produce quinones leading to oxidative stress, mtDNA damage and OXPHOS deficits. DA can also enter the mitochondria to directly inhibit the rate-limiting complex in the ETC. Upon neuronal excitation, DA is released into the synaptic cleft and reimported into presynaptic neurons or taken up by surrounding glial cells where it is degraded by COMT or MAO. DA can also be metabolized by DBH in synaptic vesicles to generate NE which exerts antioxidant effects *via* the inhibition of NOX signaling in microglia. **(C)** Serotonin (5-HT) is produced from tryptophan *via* MAO in serotonergic neurons while melatonin is produced from 5-HT in pinealocytes. 5-HT upregulates mitochondrial biogenesis, oxidative capacity and ATP synthesis and decreases oxidative stress while melatonin functions as an antioxidant and anti-inflammatory agent. Melatonin also mitigates against mitochondrial membrane permeabilization by activating Bcl-2. Once released, 5-HT can be taken up by either presynaptic neurons or glial cells *via* 5-HT transporters and broken down by MAO. In glial cells, tryptophan is oxidized *via* the KYN pathway. Astrocytes produce the neuroprotective metabolite KYNA while microglia generate the neurotoxic metabolite QUIN. Microglial immune activation in response to pro-inflammatory stimuli upregulates the expression of IDO1 to facilitate tryptophan transport into the cell, which both decreases local tryptophan availability and increases the production of QUIN in microglia. GDH, glutamate dehydrogenase; GAD, glutamate decarboxylase; GSS, glutathione synthetase; GS, glutamine synthetase; GSH, glutathione; DA, dopamine; MAO, monoamine oxidase; ROS, reactive oxygen species; mtDNA, mitochondrial DNA; OXPHOS, oxidative phosphorylation; ETC, electron transport chain; COMT, catechol-Omethyltransferase; DBH, DA betahydroxylase; NE, norepinephrine; NOX, Nicotinamide adenine dinucleotide phosphate oxidase; Bcl-2, B-cell lymphoma 2; SERT, serotonin transporter; KYN, kynurenine; KYNA, kynurenic acid; QUIN, quinolinic acid; IDO1, Indoleamine-pyrrole 2,3-dioxygenase.

Crucially, MAL functions as a mechanistic link between the molecular, neurochemical and behavioral aspects of ASC that have been highlighted here as potential contributors to psychopathology (Figure 4). Numerous reviews have comprehensively described the close coupling of mitochondrial function with glutamatergic (365–367), serotonergic (368–370) and dopaminergic (371–375) neurotransmission, although this relationship remains understudied in the context of MAL and psychopathology. Glutamatergic neurotransmission is tightly coupled to neuronal and astrocytic metabolism, and glutamate functions as a substrate for both the mitochondrial citric acid cycle and the transulfuration pathway (Figure 4A) (376, 377). In this way, glutamate fuels OXPHOS and antioxidant synthesis to promote mitochondrial function and combat oxidative stress (366, 377–380). Thus, mitochondrial metabolism is essential for glutamate-glutamine cycling, and cytoplasmic glutamate levels are regulated by mitochondrial TCA cycle flux as well as intracellular redox homeostasis (381). Dopamine (DA) auto-oxidation is also regulated by intracellular redox state (Figure 4B), and an accumulation of oxidative dopamine metabolites leads to mitochondrial membrane depolarization, decreased ETC activity and impaired ATP synthesis (374). Moreover, DA can also be directly taken up by mitochondria where it reversibly inhibits the first ETC complex, leading to oxidative stress and impaired mitochondrial energy production (373). On the other hand, DA functions as a precursor to norepinephrine (NE) which protects mitochondrial function by preventing membrane depolarization, and has both anti-inflammatory and anti-oxidant properties (382). Conversely, mitochondrial dysfunction can disrupt both glutamatergic and dopaminergic neurotransmitter signaling; TCA cycle anaplerosis diverts glutamate away from glutamine recycling, while oxidative stress promotes the generation of oxidative dopamine derivatives. Notably, the consequent shift toward glycolysis in glial cells upregulates pro-inflammatory signaling and increases the production of lactate and serine, which potentiates glutamatergic excitotoxicity (246, 383).

Similarly, a reciprocal relationship between mitochondrial function and the two branches of tryptophan metabolism modulates the synthesis of serotonin, melatonin, and kynurenine (KYN) metabolites (Figure 4C) (384). Serotonin positively regulates mitochondrial biogenesis, oxidative capacity and ATP synthesis (370, 385) while serotonin deficiency disrupts amino acid and lipid metabolism, oxidative respiration and antioxidant activity (386, 387). Serotonin functions as a precursor to melatonin, which regulates mitochondrial OXPHOS, redox homeostasis and inflammatory responses (388). Moreover, melatonin modulates the expression of the rate-limiting enzymes involved in serotonin synthesis and catabolism (389), thereby controlling the balance between the serotonin and KYN branches of the tryptophan catabolic pathway. Importantly, the KYN pathway functions as a key mediator between neuroendocrinology and neuroinflammation that is tightly regulated by interactions between glial cells and neurons. The KYN metabolites kynurenic acid (KYNA) and quinolinic acid (QUIN), respectively, activate and inhibit glutamate receptors (132). Additionally, flux in the QUIN branch of the KYN pathway that alters *de novo* NAD+ synthesis also modulates metabolic and oxidative state (390). Mitochondrial dysfunction shifts the KYN pathway toward QUIN production by increasing the demand for NAD+ (391); conversely, QUIN contributes to mitochondrial dysfunction by increasing cytoplasmic calcium concentration and superoxide production in microglia (392). A pathogenic shift toward an inflammatory microglial phenotype upregulates tryptophan import, decreasing local tryptophan availability for serotonin synthesis and further increasing the rate of QUIN production (392). Moreover, QUIN upregulates glutamatergic neurotransmission and inhibits glutamate uptake from the synaptic cleft, thereby promoting excitotoxicity (393). Altogether, a disruption of the relationship between mitochondrial function and the glutamatergic, serotonergic and dopaminergic neurotransmitter cycles increases oxidative stress and shifts glial metabolism toward glycolysis, thereby contributing to gliosis, excitotoxicity and neuroinflammation (246, 373, 393).

Thus, these neurotransmitter systems are intrinsically coupled to, and mediators of, the relationship between mitochondrial dysfunction, neuroinflammation and HPA axis signaling that is implicated in ELS and ASC. This discussion highlights that MAL plays a central role in the link between molecular stress signatures and neurophysiological- and neurochemical- mechanisms that lead to psychopathology. Moreover, these mechanisms are known to contribute to distinct neurological and behavioral aspects of ASC etiology and could function as an underlying susceptibility to allostatic load. This provides a preliminary conceptual framework for a molecular signature that may underly autistic burnout, mediate the relationship between social camouflaging and suicidality, or predispose autistic individuals to psychopathology. The interactions between environmental, psychological, physiological and molecular processes involved in ASC are notably understudied and the hypothesis proposed here remains to be further investigated. However, this model does illustrate the necessity for an interdisciplinary framework in molecular autism research. Moreover, our hypothesis describes a mechanistic interplay between MAL, HPA and neuroimmune signaling that could be an important target for future research toward improving quality of life in ASC.

## Conclusion: An integrative biopsychosocial framework for psychopathology in ASC

**Figure 5:**
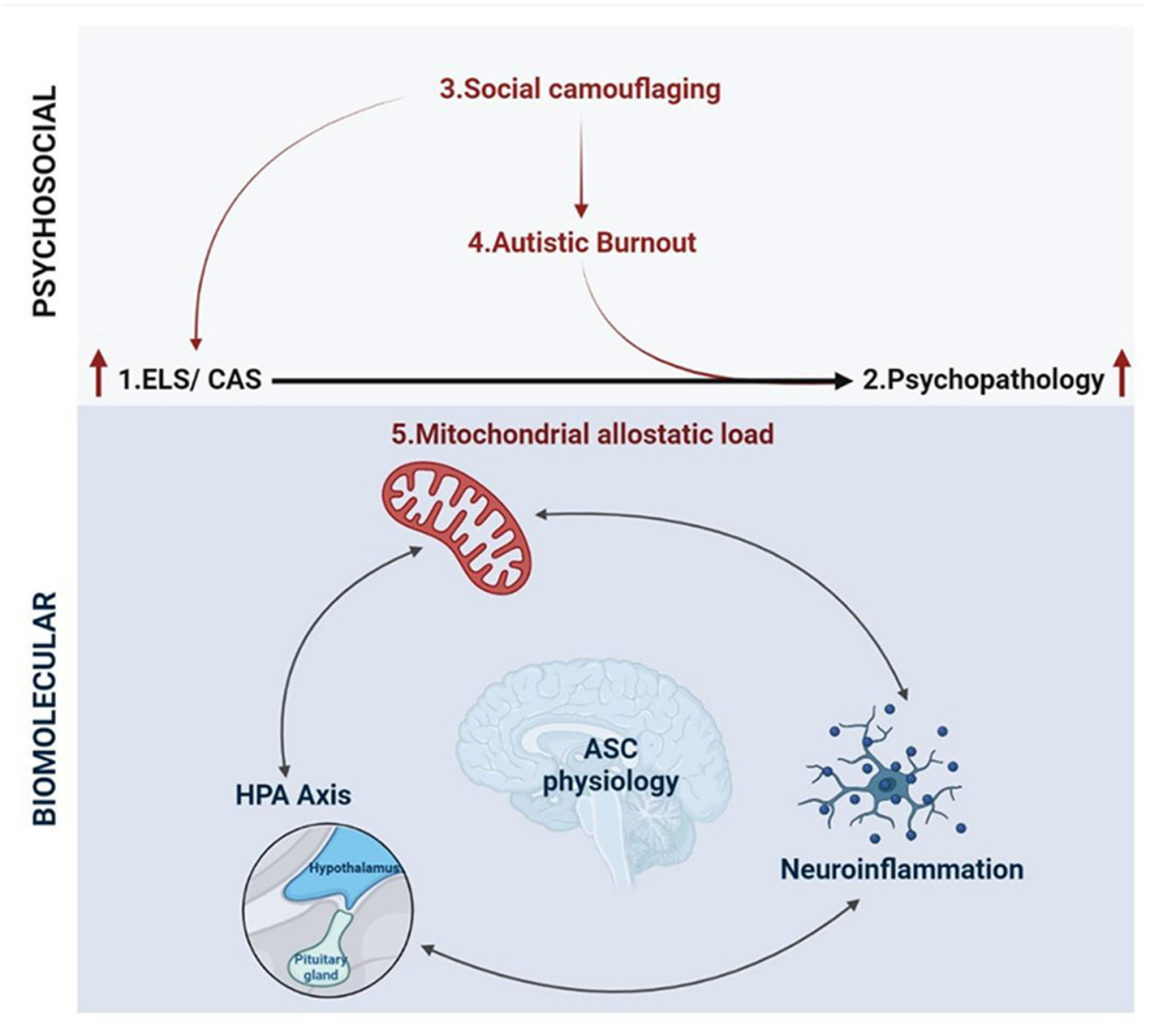
An integrative biopsychosocial vulnerability framework for the development of psychopathology in ASC (created with BioRender.com). There is a well-established relationship between exposure to early life stress (ELS) or chronic adolescent stress (CAS) and the development of psychopathology. Autism is associated with both a significantly increased risk of exposure to ELS and high rates of psychopathology and suicidality. Emerging characterizations of internal autistic experiences including social camouflaging and autistic burnout overlap closely with established definitions for ELS and lead to increased levels of anxiety, depression, self-injury and suicidality in ASC. Thus, the development of psychopathology in autism may be mediated by an increased exposure to toxic stressors culminating in allostatic overload. Molecular stress research has demonstrated that mitochondrial allostatic load (MAL) plays a central role in psychopathology following ELS by disrupting a closely coupled network between neuroimmune and neuroendocrine signaling. Cellular neuroscience has comprehensively characterized the tightly regulated biochemical relationships between (i) mitochondrial dysfunction and neuroinflammation, (ii) mitochondrial metabolism and the HPA axis, and (iii) inflammatory- and HPA-signaling. Notably, ASC etiology is also characterized by mitochondrial dysfunction, gliosis and neuroinflammation, and a complex signature of HPA axis dysregulation. Moreover, this three-way molecular interplay converges on the regulation of key neurodevelopmental processes implicated in the response to ELS and psychopathology that are also associated with distinct neurophysiological, neurochemical and behavioral aspects of ASC etiology. Collectively, this could point to a molecular vulnerability to the development of psychopathology in ASC, highlighting how psychosocial and biological factors converge to increase the risk of psychopathology and suicidality.

Molecular research into ELS is increasingly integrating various paradigms from disparate scientific disciplines in order to develop a cohesive understanding of pathological stress responses (394). While ELS research is distinctly and fundamentally separate from autism research, this discussion has highlighted the utility of such an integrative framework to shape molecular research into ASC. The hypothesis presented in this article builds on separate bodies of work, that are well-documented in their respective fields, to propose a framework for the development of psychopathology in autism (Figure 5). This framework integrates emerging literature that is foregrounding autistic experiences with the molecular model for allostatic load that quantifies the cumulative impact of biological and psychosocial stress (40). We propose that ASC is associated with both an increased exposure to, and an underlying molecular susceptibility to, ELS that contributes to allostatic overload and the subsequent development of psychopathology.

There is substantial evidence that autistic children are more likely to be exposed to ELS and psychosocial stress, while also having inadequate internal resources to cope with these stressors. The recent characterization of concepts like social camouflaging and autistic burnout further highlights that autistic children are exposed to chronic and distinct forms of stress throughout adolescence that often go unrecognized. Chronic social camouflaging creates an increasing mismatch between internal capacity and external expectations, culminating in autistic burnout. Importantly, both social camouflaging and autistic burnout are known to contribute to the development of psychopathology and suicidality in ASC. However, the physiological mechanisms that are involved in these phenomena, or their relationship to each other, remain unknown and largely unexplored by molecular researchers.

The molecular mechanisms that mediate the relationship between ELS, CAS and psychopathology revolve around interdependent signaling between mitochondrial metabolism, inflammatory immune responses and stress-responsive HPA signaling. MAL is emerging as a central modulator of the neurophysiological processes that are disrupted by ELS and contribute to psychopathology. Notably, mitochondrial dysfunction is an underlying component of ASC physiology that is sensitive to GC signaling and plays a central role in regulating HPA axis and innate immune responses. Mitochondria function as essential regulators of neurodevelopment by facilitating the metabolic shift toward OXPHOS that is required for neuronal differentiation. Mitochondria also modulate microglial activation, which is necessary for synaptogenesis and synaptic pruning during neurodevelopment. Moreover, the relationship between mitochondrial metabolism and microglial activation regulates myelin turnover, neuronal function, redox homeostasis and glial inflammatory state throughout childhood and adolescence. The HPA axis is intrinsically coupled to both mitochondrial metabolism and inflammatory signaling, and this relationship is consistently shown to be disrupted by ELS and altered in psychopathology. Notably, the serotonergic, dopaminergic and glutamatergic neurotransmitter systems that are directly involved in many psychological disorders are closely coupled to mitochondrial metabolism, modulated by the HPA axis and represent independent mechanisms that contribute to gliosis and excitotoxicity. This article has highlighted how these molecular mechanisms are implicated in the neurophysiology and neurochemistry of autism and contribute to the development of psychopathology following ELS.

Considering both the increased risk of exposure and underlying vulnerability to ELS in autism emphasizes how psychosocial and biological factors converge to increase the risk of psychopathology. This interplay could act as a novel mechanism that contributes to the high rates of depression and suicidality in autistic individuals and may reveal a biological signature that underlies autistic burnout. Autistic burnout, depression and suicidality are factors that directly impair health and wellbeing for people with autism. Much of the literature highlights the importance of improved access to accommodations, earlier diagnosis and decreased stigmatization of autistic traits in mitigating psychopathology in ASC. In addition, our framework proposes that investigating an underlying susceptibility to ELS could inform research into novel molecular interventions. Targeted therapeutic strategies could potentially protect autistic children from the development of psychopathology, facilitate recovery from autistic burnout, or identify diagnostic tools to differentiate autistic burnout from clinical depression. In particular, this framework highlights the potential to harness recent advances in mitochondrial psychobiology to better characterize the molecular determinants of mental health in autism. Ultimately, this highlights the utility of molecular research that foregrounds autistic experiences to work toward strategies that tangibly improve the quality of life of autistic individuals.

## Data availability statement

The original contributions presented in the study are included in the article/supplementary material, further inquiries can be directed to the corresponding author.

## Author contributions

CM reviewed the relevant literature, developed the conceptual framework, and wrote the paper. CO'R refined the conceptual framework, wrote the paper, coordinated project supervision, and administration and acquisition of funding. Both authors contributed to the article and approved the submitted version.

## Funding

This work was supported by the University of Cape Town and the National Research Foundation, South Africa (Grant No. 138010).

## Conflict of interest

The authors declare that the research was conducted in the absence of any commercial or financial relationships that could be construed as a potential conflict of interest.

## Publisher's note

All claims expressed in this article are solely those of the authors and do not necessarily represent those of their affiliated organizations, or those of the publisher, the editors and the reviewers. Any product that may be evaluated in this article, or claim that may be made by its manufacturer, is not guaranteed or endorsed by the publisher.

## References

[1] Whiteley P Carr K Shattock P. Research, clinical, and sociological aspects of autism. Front Psychiatry. (2021) 12:560. 10.3389/fpsyt.2021.481546 33995134 PMC8116543

[2] Milton DEM. Autistic expertise: a critical reflection on the production of knowledge in autism studies. Autism. (2014) 18:794–802. 10.1177/1362361314525281 24637428

[3] Fletcher-Watson S Adams J Brook K Charman T Crane L Cusack J. Making the future together: shaping autism research through meaningful participation. Autism. (2019) 23:943–53. 10.1177/1362361318786721 30095277 PMC6512245

[4] Brkić D Ng-Cordell E O'Brien S Scerif G Astle D Baker K. Gene functional networks and autism spectrum characteristics in young people with intellectual disability: a dimensional phenotyping study. Mol Autism. (2020) 11:98. 10.1186/s13229-020-00403-9 33308299 PMC7731560

[5] Quesnel-Vallières M Weatheritt RJ Cordes SP Blencowe BJ. Autism spectrum disorder: insights into convergent mechanisms from transcriptomics. Nat Rev Genet. (2019) 20:51–63. 10.1038/s41576-018-0066-2 30390048

[6] Wiśniowiecka-Kowalnik B Nowakowska BA. Genetics and epigenetics of autism spectrum disorder—current evidence in the field. J Appl Genet. (2019) 60:37–47. 10.1007/s13353-018-00480-w 30627967 PMC6373410

[7] Zhang XC Shu LQ Zhao X Sen Li XK. Autism spectrum disorders: autistic phenotypes and complicated mechanisms. World J Pediatr. (2019) 15:17–25. 10.1007/s12519-018-0210-2 30607884

[8] Charman T Jones CRG Pickles A Simonoff E Baird G Happé F. Defining the cognitive phenotype of autism. Brain Res. (2011) 1380:10–21. 10.1016/j.brainres.2010.10.075 21029728

[9] Berry K Russell K Frost K. Restricted and repetitive behaviors in autism spectrum disorder: a review of associated features and presentation across clinical populations. Curr Dev Disord Rep. (2018) 5:108–15. 10.1007/s40474-018-0139-0

[10] Magiati I Tay XW Howlin P. Cognitive, language, social and behavioural outcomes in adults with autism spectrum disorders: a systematic review of longitudinal follow-up studies in adulthood. Clin Psychol Rev. (2014) 34:73–86. 10.1016/j.cpr.2013.11.002 24424351

[11] Tager-Flusberg H Joseph RM. Identifying neurocognitive phenotypes in autism. Philos Trans R Soc Lond B Biol Sci. (2003) 358:303–14. 10.1098/rstb.2002.1198 12639328 PMC1201482

[12] Doyle N. Neurodiversity at work: a biopsychosocial model and the impact on working adults. Br Med Bull. (2020) 135:108. 10.1093/bmb/ldaa021 32996572 PMC7732033

[13] Bölte S De Schipper E Robison JE Wong VCN Selb M Singhal N. Classification of functioning and impairment: the development of ICF core sets for autism spectrum disorder. Autism Res. (2014) 7:167–72. 10.1002/aur.1335 24124074

[14] Leadbitter K Buckle KL Ellis C Dekker M. Autistic self-advocacy and the neurodiversity movement: implications for autism early intervention research and practice. Front Psychol. (2021) 12:782. 10.3389/fpsyg.2021.635690 33912110 PMC8075160

[15] Mesleh AG Abdulla SA El-Agnaf O. Paving the way toward personalized medicine: current advances and challenges in multi-OMICS approach in autism spectrum disorder for biomarkers discovery and patient stratification. J Pers Med. (2021) 11:41. 10.3390/jpm11010041 33450950 PMC7828397

[16] McCracken JT Anagnostou E Arango C Dawson G Farchione T Mantua V. Drug development for Autism Spectrum Disorder (ASD): progress, challenges, and future directions. Eur Neuropsychopharmacol. (2021) 48:3–31. 10.1016/j.euroneuro.2021.05.010 34158222 PMC10062405

[17] Lombardo Mv Mandelli V. Rethinking our concepts and assumptions about autism. Front Psychiatry. (2022) 13:1184. 10.3389/fpsyt.2022.903489 35722549 PMC9203718

[18] Divan G Bhavnani S Leadbitter K Ellis C Dasgupta J Abubakar A. Annual research review: achieving universal health coverage for young children with autism spectrum disorder in low- and middle-income countries: a review of reviews. J Child Psychol Psychiatry. (2021) 62:514–35. 10.1111/jcpp.13404 33905120

[19] Donohue MR Childs AW Richards M Robins DL. Race influences parent report of concerns about symptoms of autism spectrum disorder. Autism. (2019) 23:100–11. 10.1177/1362361317722030 29100475 PMC5930138

[20] Durkin MS Maenner MJ Baio J Christensen D Daniels J Fitzgerald R. Autism spectrum disorder among US children (2002-2010): socioeconomic, racial, and ethnic disparities. Am J Public Health. (2017) 107:1818–26. 10.2105/AJPH.2017.304032 28933930 PMC5637670

[21] Fombonne E Zuckerman KE. Clinical profiles of black and white children referred for autism diagnosis. J Autism Dev Disord. (2021) 52:1120–30. 10.1007/s10803-021-05019-3 33871736

[22] Hull L Petrides KV Mandy W. The female autism phenotype and camouflaging: a narrative review. Rev J Autism Dev Disord. (2020) 7:306–17. 10.1007/s40489-020-00197-9

[23] Lockwood Estrin G Milner V Spain D Happé F Colvert E. Barriers to autism spectrum disorder diagnosis for young women and girls: a systematic review. Rev J Autism Dev Disord. (2020) 8:454–70. 10.1007/s40489-020-00225-8 34868805 PMC8604819

[24] Mandell DS Wiggins LD Carpenter LA Daniels J DiGuiseppi C Durkin MS. Racial/ethnic disparities in the identification of children with autism spectrum disorders. Am J Public Health. (2009) 99:493. 10.2105/AJPH.2007.131243 19106426 PMC2661453

[25] Yingling ME Hock RM Bell BA. Time-lag between diagnosis of autism spectrum disorder and onset of publicly-funded early intensive behavioral intervention: do race-ethnicity and neighborhood matter? J Autism Dev Disord. (2018) 48:561–71. 10.1007/s10803-017-3354-3 29080927

[26] Scahill L Lord C. Subject selection and characterization in clinical trials in children with autism. CNS Spectr. (2004) 9:22–32. 10.1017/S1092852900008336 14999173

[27] Hull L Petrides KV Allison C Smith P Baron-Cohen S Lai MC. “Putting on my best normal”: social camouflaging in adults with autism spectrum conditions. J Autism Dev Disord. (2017) 47:2519–34. 10.1007/s10803-017-3166-5 28527095 PMC5509825

[28] Cage E Troxell-Whitman Z. Understanding the reasons, contexts and costs of camouflaging for autistic adults. J Autism Dev Disord. (2019) 49:1899–911. 10.1007/s10803-018-03878-x 30627892 PMC6483965

[29] Cook J Hull L Crane L Mandy W. Camouflaging in autism: a systematic review. Clin Psychol Rev. (2021) 89:102080. 10.1016/j.cpr.2021.102080 34563942

[30] Cassidy SA Gould K Townsend E Pelton M Robertson AE Rodgers J. Is Camouflaging autistic traits associated with suicidal thoughts and behaviours? Expanding the interpersonal psychological theory of suicide in an undergraduate student sample. J Autism Dev Disord. (2020) 50:3638–48. 10.1007/s10803-019-04323-3 31820344 PMC7502035

[31] Schubert-Bast S Rosenow F Klein KM Reif PS Kieslich M Strzelczyk A. The role of mTOR inhibitors in preventing epileptogenesis in patients with TSC: current evidence and future perspectives. Epilepsy Behav. (2019) 91:94–8. 10.1016/j.yebeh.2018.05.039 29941212

[32] Young N Findling RL. An update on pharmacotherapy for autism spectrum disorder in children and adolescents. Curr Opin Psychiatry. (2015) 28:91–101. 10.1097/YCO.0000000000000132 25602248

[33] Vorstman JAS Parr JR Moreno-De-Luca D Anney RJL Nurnberger JI Hallmayer JF. Autism genetics: opportunities and challenges for clinical translation. Nat Rev Genet. (2017) 18:362–76. 10.1038/nrg.2017.4 28260791

[34] Jeste SS Geschwind DH. Clinical trials for neurodevelopmental disorders: at a therapeutic frontier. Sci Transl Med. (2016) 8:321fs1 10.1126/scitranslmed.aad9874 26764154

[35] Loth E Murphy DG Spooren W. Defining precision medicine approaches to autism spectrum disorders: concepts and challenges. Front Psychiatry. (2016) 7:188. 10.3389/fpsyt.2016.00188 27965598 PMC5126086

[36] Sestan N State MW. Lost in translation: traversing the complex path from genomics to therapeutics in autism spectrum disorder. Neuron. (2018) 100:406–23. 10.1016/j.neuron.2018.10.015 30359605 PMC6989093

[37] Singletary WM. An integrative model of autism spectrum disorder: ASD as a neurobiological disorder of experienced environmental deprivation, early life stress and allostatic overload. Neuropsychoanalysis. (2015) 17:81–119. 10.1080/15294145.2015.1092334

[38] Scarpa A Swain DM Factor RS Dahiya A v Bertollo JR. Enhancing flexibility: a biosocial model for resilience to adversity in youth with autism. Sage Open. (2021) 11:21582440211037997. 10.1177/21582440211037997

[39] Makris G Eleftheriades A Pervanidou P. Early life stress, hormones, and neurodevelopmental disorders. Horm Res Paediatr. (2022) 1–8. 10.1159/000523942. [Epub ahead of print]. 35259742

[40] Picard M McEwen BS. Psychological stress and mitochondria: a conceptual framework. Psychosom Med. (2018) 80:126. 10.1097/PSY.0000000000000544 29389735 PMC5901651

[41] Etyemez S Saleh A Hamilton JE Koshy AJ Abraham JE Selek S. Higher prevalence of mood disorders in admitted patients with autism. Neurol Psychiatry Brain Res. (2020) 37:87–90. 10.1016/j.npbr.2020.06.00725575287

[42] Smith IC White SW. Socio-emotional determinants of depressive symptoms in adolescents and adults with autism spectrum disorder: a systematic review. Autism. (2020) 24:995–1010. 10.1177/1362361320908101 32191120

[43] Oakley B Loth E Murphy DG. Autism and mood disorders. Int Rev Psychiatry. (2021) 33:280–99. 10.1080/09540261.2021.1872506 33648430

[44] Costa AP Loor C Steffgen G. Suicidality in adults with autism spectrum disorder: the role of depressive symptomatology, alexithymia, and antidepressants. J Autism Dev Disord. (2020) 50:3585–97. 10.1007/s10803-020-04433-3 32172508

[45] Dow D Morgan L Hooker JL Michaels MS Joiner TE Woods J. Anxiety, depression, and the interpersonal theory of suicide in a community sample of adults with autism spectrum disorder. Arch Suicide Res. (2021) 25:297–314. 10.1080/13811118.2019.1678537 31656121

[46] Muscatello RA Andujar J Taylor JL Corbett BA. Exploring key physiological system profiles at rest and the association with depressive symptoms in autism spectrum disorder. J Autism Dev Disord. (2020) 51:15–29. 10.1007/s10803-020-04516-1 32350791 PMC7606213

[47] Kõlves K Fitzgerald C Nordentoft M Wood SJ Erlangsen A. Assessment of suicidal behaviors among individuals with autism spectrum disorder in Denmark. JAMA Netw Open. (2021) 4:e2033565. 10.1001/jamanetworkopen.2020.33565 33433599 PMC12578491

[48] Cassidy S Bradley P Robinson J Allison C McHugh M Baron-Cohen S. Suicidal ideation and suicide plans or attempts in adults with Asperger's syndrome attending a specialist diagnostic clinic: a clinical cohort study. Lancet Psychiatry. (2014) 1:142–7. 10.1016/S2215-0366(14)70248-2 26360578

[49] Howe SJ Hewitt K Baraskewich J Cassidy S McMorris CA. Suicidality among children and youth with and without autism spectrum disorder: a systematic review of existing risk assessment tools. J Autism Dev Disord. (2020) 50:3462–76. 10.1007/s10803-020-04394-7 32100237

[50] Haruvi-Lamdan N Horesh D Golan O. PTSD and autism spectrum disorder: co-morbidity, gaps in research, and potential shared mechanisms. Psychol Trauma. (2018) 10:290–9. 10.1037/tra0000298 28726442

[51] South M Beck JS Lundwall R Christensen M Cutrer EA Gabrielsen TP. Unrelenting depression and suicidality in women with autistic traits. J Autism Dev Disord. (2020) 50:3606–19. 10.1007/s10803-019-04324-2 31820343

[52] Warrier V Baron-Cohen S. Childhood trauma, life-time self-harm, and suicidal behaviour and ideation are associated with polygenic scores for autism. Mol Psychiatry. (2021) 26:1670–84. 10.1038/s41380-019-0550-x 31659270 PMC8159746

[53] Unruh KE Bodfish JW Gotham KO. Adults with autism and adults with depression show similar attentional biases to social-affective images. J Autism Dev Disord. (2020) 50:2336–47. 10.1007/s10803-018-3627-5 29882107 PMC6286233

[54] Williams ZJ. Commentary: the construct validity of ‘camouflaging' in autism: psychometric considerations and recommendations for future research - reflection on Lai et al. (2020). J Child Psychol Psychiatry. (2022) 63:118–21. 10.1111/jcpp.13468 34145574 PMC8678389

[55] Hull L Mandy W Lai MC Baron-Cohen S Allison C Smith P. Development and validation of the camouflaging autistic traits questionnaire (CAT-Q). J Autism Dev Disord. (2019) 49:819–33. 10.1007/s10803-018-3792-6 30361940 PMC6394586

[56] Lai MC Lombardo M v Ruigrok ANV Chakrabarti B Auyeung B Szatmari P. Quantifying and exploring camouflaging in men and women with autism. Autism. (2017) 21:690–702. 10.1177/1362361316671012 27899710 PMC5536256

[57] Lai MC Hull L Mandy W Chakrabarti B Nordahl CW Lombardo MV. Commentary: ‘Camouflaging' in autistic people – reflection on Fombonne (2020). J Child Psychol Psychiatry. (2021) 62:1037–41. 10.1111/jcpp.13344 33289092

[58] Livingston LA Happé F. Conceptualising compensation in neurodevelopmental disorders: reflections from autism spectrum disorder. Neurosci Biobehav Rev. (2017) 80:729–42. 10.1016/j.neubiorev.2017.06.005 28642070 PMC7374933

[59] Raymaker DM Teo AR Steckler NA Lentz B Scharer M Santos AD. “Having all of your internal resources exhausted beyond measure and being left with no clean-up crew”: defining autistic burnout. Autism Adulthood. (2020) 2:132–43. 10.1089/aut.2019.0079 32851204 PMC7313636

[60] Higgins JM Arnold SRC Weise J Pellicano E Trollor JN. Defining autistic burnout through experts by lived experience: Grounded Delphi method investigating #AutisticBurnout. Autism. (2021) 25:2356–69. 10.1177/13623613211019858 34088219

[61] Mantzalas J Richdale AL Adikari A Lowe J Dissanayake C. What is autistic burnout? A thematic analysis of posts on two online platforms. Autism Adulthood. (2021) 4:52–65. 10.1089/aut.2021.0021 PMC8992925 36605565

[62] Mantzalas J Richdale AL Dissanayake C. A conceptual model of risk and protective factors for autistic burnout. Autism Res. (2022) 15:976–87. 10.1002/aur.2722 35416430

[63] Bradley L Shaw R Baron-Cohen S Cassidy S. Autistic adults' experiences of camouflaging and its perceived impact on mental health. Autism Adulthood. (2021) 3:320–9. 10.1089/aut.2020.0071 PMC8992917 36601637

[64] Leedham A Thompson AR Smith R Freeth M. ‘I was exhausted trying to figure it out': the experiences of females receiving an autism diagnosis in middle to late adulthood. Autism. (2020) 24:135–46. 10.1177/1362361319853442 31144507

[65] Tubío-Fungueiriño M Cruz S Sampaio A Carracedo A Fernández-Prieto M. Social camouflaging in females with autism spectrum disorder: a systematic review. J Autism Dev Disord. (2021) 51:2190–9. 10.1007/s10803-020-04695-x 32926304

[66] Kim SY Lecavalier L. Depression in young autistic people: a scoping review. Res Autism Spectr Disord. (2021) 88:101841. 10.1016/j.rasd.2021.101841

[67] Hull L Levy L Lai MC Petrides KV Baron-Cohen S Allison C. Is social camouflaging associated with anxiety and depression in autistic adults? Mol Autism. (2021) 12:1–13. 10.1186/s13229-021-00421-1 33593423 PMC7885456

[68] Montero-Marín J García-Campayo J Mera DM Del Hoyo YL. A new definition of burnout syndrome based on Farber's proposal. J Occupational Med Toxicol. (2009) 4:1–17. 10.1186/1745-6673-4-31 19948055 PMC2794272

[69] Lilley R Lawson W Hall G Mahony J Clapham H Heyworth M. “A way to be me”: autobiographical reflections of autistic adults diagnosed in mid-to-late adulthood. Autism. (2021) 26:1395–408. 10.1177/13623613211050694 34674564

[70] Stagg SD Belcher H. Living with autism without knowing: receiving a diagnosis in later life. Health Psychol Behav Med. (2019) 7:348–61. 10.1080/21642850.2019.1684920 34040855 PMC8114403

[71] van der Cruijsen R Boyer BE. Explicit and implicit self-esteem in youth with autism spectrum disorders. Autism. (2021) 25:349–60. 10.1177/1362361320961006 33054401 PMC7874369

[72] Malinovskaya NA Morgun A V Lopatina OL Panina YA Volkova VV Gasymly EL. Early life stress: consequences for the development of the brain. Neurosci Behav Physiol. (2018) 48:233–50. 10.1007/s11055-018-0557-911168837

[73] Sheth C McGlade E Yurgelun-Todd D. Chronic stress in adolescents and its neurobiological and psychopathological consequences: an RDoC perspective. Chronic Stress. (2017) 1:2470547017715645. 10.1177/2470547017715645 29527590 PMC5841253

[74] Fogelman N Canli T. Early life stress, physiology, and genetics: a review. Front Psychol. (2019) 10:1668. 10.3389/fpsyg.2019.01668 31428006 PMC6688564

[75] Lähdepuro A Savolainen K Lahti-Pulkkinen M Eriksson JG Lahti J Tuovinen S. The impact of early life stress on anxiety symptoms in late adulthood. Sci Rep. (2019) 9:1–13. 10.1038/s41598-019-40698-0 30867476 PMC6416302

[76] LeMoult J Humphreys KL Tracy A Hoffmeister JA Ip E Gotlib IH. Meta-analysis: exposure to early life stress and risk for depression in childhood and adolescence. J Am Acad Child Adolesc Psychiatry. (2020) 59:842–55. 10.1016/j.jaac.2019.10.011 31676392 PMC11826385

[77] Sahle BW Reavley NJ Li W Morgan AJ Yap MBH Reupert A. The association between adverse childhood experiences and common mental disorders and suicidality: an umbrella review of systematic reviews and meta-analyses. Eur Child Adolesc Psychiatry. (2021) 1:1–11. 10.1007/s00787-021-01745-2 33638709

[78] Su YY D'Arcy C Li M O'Donnell KJ Caron J Meaney MJ. Specific and cumulative lifetime stressors in the aetiology of major depression: a longitudinal community-based population study. Epidemiol Psychiatr Sci. (2022) 31:e3. 10.1017/S2045796021000779 35078547 PMC8851045

[79] Targum SD Nemeroff CB. The effect of early life stress on adult psychiatric disorders. Innov Clin Neurosci. (2019) 16:35–7. 31037228 PMC6450674

[80] Jones CM Merrick MT Houry DE. Identifying and preventing adverse childhood experiences: implications for clinical practice. JAMA. (2020) 323:25–6. 10.1001/jama.2019.18499 31689343 PMC9173206

[81] Agorastos A Pervanidou P Chrousos GP Baker DG. Developmental trajectories of early life stress and trauma: a narrative review on neurobiological aspects beyond stress system dysregulation. Front Psychiatry. (2019) 10:118. 10.3389/fpsyt.2019.00118 30914979 PMC6421311

[82] Picard M McEwen BS Epel ES Sandi C. An energetic view of stress: focus on mitochondria. Front Neuroendocrinol. (2018) 49:72–85. 10.1016/j.yfrne.2018.01.001 29339091 PMC5964020

[83] Kuenzel E Seguin D Nicolson R Duerden EG. Early adversity and positive parenting: association with cognitive outcomes in children with autism spectrum disorder. Autism Res. (2021) 14:2654–62. 10.1002/aur.2613 34549545

[84] Griffiths S Allison C Kenny R Holt R Smith P Baron-Cohen S. The vulnerability experiences quotient (VEQ): a study of vulnerability, mental health and life satisfaction in autistic adults. Autism Res. (2019) 12:1516–28. 10.1002/aur.2162 31274233 PMC6851759

[85] Schroeder JH Cappadocia MC Bebko JM Pepler DJ Weiss JA. Shedding light on a pervasive problem: a review of research on bullying experiences among children with autism spectrum disorders. J Autism Dev Disord. (2014) 44:1520–34. 10.1007/s10803-013-2011-8 24464616

[86] Trundle G Jones KA Ropar D Egan V. Prevalence of victimisation in autistic individuals: a systematic review and meta-analysis. Trauma Violence Abuse. (2022) 152483802210936. 10.1177/15248380221093689. [Epub ahead of print]. 35524162 PMC10486169

[87] Tierney S Burns J Kilbey E. Looking behind the mask: social coping strategies of girls on the autistic spectrum. Res Autism Spectr Disord. (2016) 23:73–83. 10.1016/j.rasd.2015.11.013

[88] Cage E Di Monaco J Newell V. Experiences of autism acceptance and mental health in autistic adults. J Autism Dev Disord. (2018) 48:473–84. 10.1007/s10803-017-3342-7 29071566 PMC5807490

[89] Markus HJ Nurius PS. “Self-understanding and self-regulation in middle childhood. In: Collins WA editor. Development During Middle Childhood: The Years From Six to Twelve. Washington DC: National Academies Press (US) (1984). Available online at: https://www.ncbi.nlm.nih.gov/books/NBK216782/25032422

[90] Cheroni C Caporale N Testa G. Autism spectrum disorder at the crossroad between genes and environment: contributions, convergences, and interactions in ASD developmental pathophysiology. Molecular Autism. (2020) 11:69. 10.1186/s13229-020-00370-1 32912338 PMC7488083

[91] Juster RP McEwen BS Lupien SJ. Allostatic load biomarkers of chronic stress and impact on health and cognition. Neurosci Biobehav Rev. (2010) 35:2–16. 10.1016/j.neubiorev.2009.10.002 19822172

[92] McEwen CA. Connecting the biology of stress, allostatic load and epigenetics to social structures and processes. Neurobiol Stress. (2022) 17:100426. 10.1016/j.ynstr.2022.100426 35535261 PMC9076953

[93] Allen J Caruncho HJ Kalynchuk LE. Severe life stress, mitochondrial dysfunction, and depressive behavior: a pathophysiological and therapeutic perspective. Mitochondrion. (2021) 56:111–7. 10.1016/j.mito.2020.11.010 33220501

[94] Guidi J Lucente M Sonino N Fava GA. Allostatic load and its impact on health: a systematic review. Psychother Psychosom. (2021) 90:11–27. 10.1159/000510696 32799204

[95] Taylor JL Corbett BA. A review of rhythm and responsiveness of cortisol in individuals with autism spectrum disorders. Psychoneuroendocrinology. (2014) 49:207–28. 10.1016/j.psyneuen.2014.07.015 25108163 PMC4165710

[96] Daniels TE Olsen EM Tyrka AR. Stress and psychiatric disorders: the role of mitochondria. Annu Rev Clin Psychol. (2020) 16:165–86. 10.1146/annurev-clinpsy-082719-104030 32092280 PMC8007172

[97] Picard M Trumpff C Burelle Y. Mitochondrial psychobiology: foundations and applications. Curr Opin Behav Sci. (2019) 28:142–51. 10.1016/j.cobeha.2019.04.015 32637466 PMC7339630

[98] Hoffmann A Spengler D. The mitochondrion as potential interface in early-life stress brain programming. Front Behav Neurosci. (2018) 12:306. 10.3389/fnbeh.2018.00306 30574076 PMC6291450

[99] Boeck C Koenig AM Schury K Geiger ML Karabatsiakis A Wilker S. Inflammation in adult women with a history of child maltreatment: the involvement of mitochondrial alterations and oxidative stress. Mitochondrion. (2016) 30:197–207. 10.1016/j.mito.2016.08.006 27530300

[100] Gumpp AM Behnke A Ramo-Fernández L Radermacher P Gündel H Ziegenhain U. Investigating mitochondrial bioenergetics in peripheral blood mononuclear cells of women with childhood maltreatment from post-parturition period to one-year follow-up. Psychol Med. (2022). 10.1017/S0033291722000411 [Epub ahead of print]. 35311632 PMC10317795

[101] Tyrka AR Parade SH Price LH Kao HT Porton B Philip NS. Alterations of mitochondrial DNA copy number and telomere length with early adversity and psychopathology. Biol Psychiatry. (2016) 79:78–86. 10.1016/j.biopsych.2014.12.025 25749099 PMC4503518

[102] Ridout KK Khan M Ridout SJ. Adverse childhood experiences run deep: toxic early life stress, telomeres, and mitochondrial DNA copy number, the biological markers of cumulative stress. BioEssays. (2018) 40:1800077. 10.1002/bies.201800077 30067291

[103] Trumpff C Marsland AL Basualto-Alarcón C Martin JL Carroll JE Sturm G. Acute psychological stress increases serum circulating cell-free mitochondrial DNA. Psychoneuroendocrinology. (2019) 106:268–76. 10.1016/j.psyneuen.2019.03.026 31029929 PMC6589121

[104] Trumpff C Michelson J Lagranha CJ Taleon V Karan KR Sturm G. Stress and circulating cell-free mitochondrial DNA: a systematic review of human studies, physiological considerations, and technical recommendations. Mitochondrion. (2021) 59:225–45. 10.1016/j.mito.2021.04.002 33839318 PMC8418815

[105] do Prado CH Grassi-Oliveira R Wieck A Zaparte A Filho LD da Silva Morrone M. The impact of childhood maltreatment on redox state: relationship with oxidative damage and antioxidant defenses in adolescents with no psychiatric disorder. Neurosci Lett. (2016) 617:173–7. 10.1016/j.neulet.2016.01.062 26845563

[106] Silva RC Maffioletti E Gennarelli M Baune BT Minelli A. Biological correlates of early life stressful events in major depressive disorder. Psychoneuroendocrinology. (2021) 125:105103. 10.1016/j.psyneuen.2020.105103 33360031

[107] Sahafi E Peeri M Hosseini MJ Azarbyjani MA. Cardiac oxidative stress following maternal separation stress was mitigated following adolescent voluntary exercise in adult male rat. Physiol Behav. (2018) 183:39–45. 10.1016/j.physbeh.2017.10.022 29061441

[108] Réus GZ Fernandes GC de Moura AB Silva RH Darabas AC de Souza TG. Early life experience contributes to the developmental programming of depressive-like behaviour, neuroinflammation and oxidative stress. J Psychiatr Res. (2017) 95:196–207. 10.1016/j.jpsychires.2017.08.020 28886447

[109] Ruigrok SR Yim K Emmerzaal TL Geenen B Stöberl N den Blaauwen JL. Effects of early-life stress on peripheral and central mitochondria in male mice across ages. Psychoneuroendocrinology. (2021) 132:105346. 10.1016/j.psyneuen.2021.105346 34274734

[110] Picard M McEwen BS. Psychological stress and mitochondria: a systematic review. Psychosom Med. (2018) 80:141. 10.1097/PSY.0000000000000545 29389736 PMC5901654

[111] Tobe EH. Mitochondrial dysfunction, oxidative stress, and major depressive disorder. Neuropsychiatr Dis Treat. (2013) 9:567. 10.2147/NDT.S44282 23650447 PMC3640606

[112] Bansal Y Kuhad A. Mitochondrial dysfunction in depression. Curr Neuropharmacol. (2016) 14:610–8. 10.2174/1570159X14666160229114755 26923778 PMC4981740

[113] Allen J Romay-Tallon R Brymer KJ Caruncho HJ Kalynchuk LE. Mitochondria and mood: mitochondrial dysfunction as a key player in the manifestation of depression. Front Neurosci. (2018) 12:386. 10.3389/fnins.2018.00386 29928190 PMC5997778

[114] Xie X Shen Q Yu C Xiao Q Zhou J Xiong Z. Depression-like behaviors are accompanied by disrupted mitochondrial energy metabolism in chronic corticosterone-induced mice. J Steroid Biochem Mol Biol. (2020) 200:105607. 10.1016/j.jsbmb.2020.105607 32045672

[115] Głombik K Budziszewska B Basta-Kaim A. Mitochondria-targeting therapeutic strategies in the treatment of depression. Mitochondrion. (2021) 58:169–78. 10.1016/j.mito.2021.03.006 33766747

[116] Kolar D Kleteckova L Brozka H Vales K. Mini-review: brain energy metabolism and its role in animal models of depression, bipolar disorder, schizophrenia and autism. Neurosci Lett. (2021) 760:136003. 10.1016/j.neulet.2021.136003 34098028

[117] Rappeneau V Wilmes L Touma C. Molecular correlates of mitochondrial dysfunctions in major depression: evidence from clinical and rodent studies. Mol Cell Neurosci. (2020) 109:103555. 10.1016/j.mcn.2020.103555 32979495

[118] Chung JK Lee SY Park M Joo EJ Kim SA. Investigation of mitochondrial DNA copy number in patients with major depressive disorder. Psychiatry Res. (2019) 282:112616. 10.1016/j.psychres.2019.112616 31639552

[119] Cai N Chang S Li Y Li Q Hu J Liang J. Molecular signatures of major depression. Curr Biol. (2015) 25:1146–56. 10.1016/j.cub.2015.03.008 25913401 PMC4425463

[120] Ben-Shachar D Karry R. Neuroanatomical pattern of mitochondrial complex I pathology varies between schizophrenia, bipolar disorder and major depression. PLoS ONE. (2008) 3:e3676. 10.1371/journal.pone.0003676 18989376 PMC2579333

[121] Anderson G. Linking the biological underpinnings of depression: role of mitochondria interactions with melatonin, inflammation, sirtuins, tryptophan catabolites, DNA repair and oxidative and nitrosative stress, with consequences for classification and cognition. Prog Neuropsychopharmacol Biol Psychiatry. (2018) 80:255–66. 10.1016/j.pnpbp.2017.04.022 28433458

[122] Karabatsiakis A Böck C Salinas-Manrique J Kolassa S Calzia E Dietrich DE. Mitochondrial respiration in peripheral blood mononuclear cells correlates with depressive subsymptoms and severity of major depression. Transl Psychiatry. (2014) 4:e397. 10.1038/tp.2014.44 26126180 PMC4080325

[123] Zugno AI Pacheco FD Budni J de Oliveira MB Canever L Heylmann AS. Maternal deprivation disrupts mitochondrial energy homeostasis in the brain of rats subjected to ketamine-induced schizophrenia. Metab Brain Dis. (2015) 30:1043–53. 10.1007/s11011-015-9671-3 25920483

[124] Kambe Y Miyata A. Potential involvement of the mitochondrial unfolded protein response in depressive-like symptoms in mice. Neurosci Lett. (2015) 588:166–71. 10.1016/j.neulet.2015.01.006 25576703

[125] Ortmann CF Réus GZ Ignácio ZM Abelaira HM Titus SE de Carvalho P. Enriched flavonoid fraction from Cecropia pachystachya Trécul leaves exerts antidepressant-like behavior and protects brain against oxidative stress in rats subjected to chronic mild stress. Neurotox Res. (2016) 29:469–83. 10.1007/s12640-016-9596-6 26762362

[126] Sonei N Amiri S Jafarian I Anoush M Rahimi-Balaei M Bergen H. Mitochondrial dysfunction bridges negative affective disorders and cardiomyopathy in socially isolated rats: pros and cons of fluoxetine. World J Biol Psychiatry. (2016) 18:39–53. 10.3109/15622975.2016.1149218 27031288

[127] Martín-Aragón S Villar Á Benedí J. Age-dependent effects of esculetin on mood-related behavior and cognition from stressed mice are associated with restoring brain antioxidant status. Prog Neuropsychopharmacol Biol Psychiatry. (2016) 65:1–16. 10.1016/j.pnpbp.2015.08.007 26290950

[128] Rinwa P Kumar A. Piperine potentiates the protective effects of curcumin against chronic unpredictable stress-induced cognitive impairment and oxidative damage in mice. Brain Res. (2012) 1488:38–50. 10.1016/j.brainres.2012.10.002 23099054

[129] Nollet M Hicks H McCarthy AP Wu H Möller-Levet CS Laing EE. REM sleep's unique associations with corticosterone regulation, apoptotic pathways, and behavior in chronic stress in mice. Proc Natl Acad Sci USA. (2019) 116:2733–42. 10.1073/pnas.1816456116 30683720 PMC6377491

[130] Bagot RCC Cates HMM Purushothaman I Lorsch ZSS Walker DMM Wang J. Circuit-wide transcriptional profiling reveals brain region-specific gene networks regulating depression susceptibility. Neuron. (2016) 90:969–83. 10.1016/j.neuron.2016.04.015 27181059 PMC4896746

[131] von Ziegler LM Floriou-Servou A Waag R Das Gupta RR Sturman O Gapp K. Multiomic profiling of the acute stress response in the mouse hippocampus. Nat Commun. (2022) 13:1–20. 10.1038/s41467-022-29367-5 35383160 PMC8983670

[132] Lombard J. Autism: a mitochondrial disorder? Med Hypotheses. (1998) 50:497–500. 10.1016/S0306-9877(98)90270-5 9710323

[133] Siddiqui MF Elwell C Johnson MH. Mitochondrial dysfunction in autism spectrum disorders. Autism Open Access. (2016) 6:1000190. 10.4172/2165-7890.1000190 27928515 PMC5137782

[134] Griffiths KK Levy RJ. Evidence of mitochondrial dysfunction in autism: biochemical links, genetic-based associations, and non-energy-related mechanisms. Oxid Med Cell Longev. (2017) 2017:4314025. 10.1155/2017/4314025 28630658 PMC5467355

[135] Rose S Niyazov DM Rossignol DA Goldenthal M Kahler SG Frye RE. Clinical and molecular characteristics of mitochondrial dysfunction in autism spectrum disorder. Mol Diagnosis Therapy. (2018) 22:571–93. 10.1007/s40291-018-0352-x 30039193 PMC6132446

[136] Varga NÁ Pentelényi K Balicza P Gézsi A Reményi V Hársfalvi V. Mitochondrial dysfunction and autism: comprehensive genetic analyses of children with autism and mtDNA deletion. Behav Brain Functions. (2018) 14:1–4. 10.1186/s12993-018-0135-x 29458409 PMC5819172

[137] Castora FJ. Mitochondrial function and abnormalities implicated in the pathogenesis of ASD. Prog Neuropsychopharmacol Biol Psychiatry. (2019) 92:83–108. 10.1016/j.pnpbp.2018.12.015 30599156

[138] Citrigno L Muglia M Qualtieri A Spadafora P Cavalcanti F Pioggia G. The mitochondrial dysfunction hypothesis in autism spectrum disorders: current status and future perspectives. Int J Mol Sci. (2020) 21:5785. 10.3390/ijms21165785 32806635 PMC7461038

[139] Frye RE. Mitochondrial dysfunction in autism spectrum disorder: unique abnormalities and targeted treatments. Semin Pediatr Neurol. (2020) 35:100829. 10.1016/j.spen.2020.100829 32892956

[140] Giulivi C Zhang YF Omanska-Klusek A Ross-Inta C Wong S Hertz-Picciotto I. Mitochondrial dysfunction in autism. JAMA. (2010) 304:2389–96. 10.1001/jama.2010.1706 21119085 PMC3915058

[141] Valiente-Pallejà A Torrell H Muntané G Cortés MJ Martínez-Leal R Abasolo N. Genetic and clinical evidence of mitochondrial dysfunction in autism spectrum disorder and intellectual disability. Hum Mol Genet. (2018) 27:891–900. 10.1093/hmg/ddy009 29340697

[142] Gu F Chauhan V Kaur K Brown WT LaFauci G Wegiel J. Alterations in mitochondrial DNA copy number and the activities of electron transport chain complexes and pyruvate dehydrogenase in the frontal cortex from subjects with autism. Transl Psychiatry. (2013) 3:e299. 10.1038/tp.2013.68 24002085 PMC3784762

[143] Chen S Li Z He Y Zhang F Li H Liao Y. Elevated mitochondrial DNA copy number in peripheral blood cells is associated with childhood autism. BMC Psychiatry. (2015) 15:50. 10.1186/s12888-015-0432-y 25884388 PMC4367837

[144] Weissman JR Kelley RI Bauman ML Cohen BH Murray KF Mitchell RL. Mitochondrial disease in autism spectrum disorder patients: a cohort analysis. PLoS ONE. (2008) 3:e3815. 10.1371/journal.pone.0003815 19043581 PMC2584230

[145] Rossignol DA Frye RE. Mitochondrial dysfunction in autism spectrum disorders: a systematic review and meta-analysis. Mol Psychiatry. (2012) 17:290–314. 10.1038/mp.2010.136 21263444 PMC3285768

[146] Tang G Gutierrez Rios P Kuo SH Akman HO Rosoklija G Tanji K. Mitochondrial abnormalities in temporal lobe of autistic brain. Neurobiol Dis. (2013) 54:349–61. 10.1016/j.nbd.2013.01.006 23333625 PMC3959772

[147] Khemakhem AM Frye RE El-Ansary A Al-Ayadhi L ben Bacha A. Novel biomarkers of metabolic dysfunction is autism spectrum disorder: potential for biological diagnostic markers. Metab Brain Dis. (2017) 32:1983–97. 10.1007/s11011-017-0085-2 28831647

[148] Hassan H Zakaria F Makpol S Karim NA. A link between mitochondrial dysregulation and idiopathic autism spectrum disorder (ASD): alterations in mitochondrial respiratory capacity and membrane potential. Curr Issues Mol Biol. (2021) 43:2238–52. 10.3390/cimb43030157 34940131 PMC8928939

[149] Pecorelli A Ferrara F Messano N Cordone V Schiavone ML Cervellati F. Alterations of mitochondrial bioenergetics, dynamics, and morphology support the theory of oxidative damage involvement in autism spectrum disorder. FASEB J. (2020) 34:6521–38. 10.1096/fj.201902677R 32246805

[150] Frye RE Lionnard L Singh I Karim MA Chajra H Frechet M. Mitochondrial morphology is associated with respiratory chain uncoupling in autism spectrum disorder. Transl Psychiatry. (2021) 11:527. 10.1038/s41398-021-01647-6 34645790 PMC8514530

[151] Ginsberg MR Rubin RA Falcone T Ting AH Natowicz MR. Brain transcriptional and epigenetic associations with autism. PLoS ONE. (2012) 7:e44736. 10.1371/journal.pone.0044736 22984548 PMC3440365

[152] Zeidán-Chuliá F de Oliveira BHN Salmina AB Casanova MF Gelain DP Noda M. Altered expression of Alzheimer's disease-related genes in the cerebellum of autistic patients: a model for disrupted brain connectome and therapy. Cell Death Dis. (2014) 5:e1250. 10.1038/cddis.2014.227 24853428 PMC4047885

[153] Aldosary M Al-Bakheet A Al-Dhalaan H Almass R Alsagob M Al-Younes B. Rett syndrome, a neurodevelopmental disorder, whole-transcriptome, and mitochondrial genome multiomics analyses identify novel variations and disease pathways. OMICS. (2020) 24:160–71. 10.1089/omi.2019.0192 32105570

[154] Forés-Martos J Catalá-López F Sánchez-Valle J Ibáñez K Tejero H Palma-Gudiel H. Transcriptomic metaanalyses of autistic brains reveals shared gene expression and biological pathway abnormalities with cancer. Mol Autism. (2019) 10:17. 10.1186/s13229-019-0262-8 31007884 PMC6454734

[155] Mahony C O'ryan C. Convergent canonical pathways in autism spectrum disorder from proteomic, transcriptomic and DNA methylation data. Int J Mol Sci. (2021) 22:10757. 10.3390/ijms221910757 34639097 PMC8509728

[156] Bjørklund G Meguid NA El-Bana MA Tinkov AA Saad K Dadar M. Oxidative stress in autism spectrum disorder. Mol Neurobiol. (2020) 57:2314–32. 10.1007/s12035-019-01742-2 32026227

[157] Hu T Dong Y He C Zhao M He Q. The gut microbiota and oxidative stress in autism spectrum disorders (ASD). Oxid Med Cell Longev. (2020) 2020:8396708. 10.1155/2020/8396708 33062148 PMC7547345

[158] Chen L Shi XJ Liu H Mao X Gui LN Wang H. Oxidative stress marker aberrations in children with autism spectrum disorder: a systematic review and meta-analysis of 87 studies (N = 9109). Transl Psychiatry. (2021) 11:1–10. 10.1038/s41398-020-01135-3 33414386 PMC7791110

[159] Manivasagam T Arunadevi S Essa MM SaravanaBabu C Borah A Thenmozhi AJ. Role of oxidative stress and antioxidants in autism. Adv Neurobiol. (2020) 24:193–206. 10.1007/978-3-030-30402-7_7 32006361

[160] Pangrazzi L Balasco L Bozzi Y. Oxidative stress and immune system dysfunction in autism spectrum disorders. Int J Mol Sci. (2020) 21:3293. 10.3390/ijms21093293 32384730 PMC7247582

[161] Thorsen M. Oxidative stress, metabolic and mitochondrial abnormalities associated with autism spectrum disorder. Progress Mol Biol Transl Sci. (2020) 173:331–54. 10.1016/bs.pmbts.2020.04.018 32711815

[162] Ceruso A Martínez-Cengotitabengoa M Peters-Corbett A Diaz-Gutierrez MJ Martínez-Cengotitabengoa M. Alterations of the HPA axis observed in patients with major depressive disorder and their relation to early life stress: a systematic review. Neuropsychobiology. (2020) 79:417–27. 10.1159/000506484 32203965

[163] Gandal MJ Haney JR Parikshak NN Leppa V Ramaswami G Hartl C. Shared molecular neuropathology across major psychiatric disorders parallels polygenic overlap. Science. (2018) 359:693. 10.1126/science.aad6469 32015716 PMC6996074

[164] Pervanidou P Chrousos GP. Early-life stress: from neuroendocrine mechanisms to stress-related disorders. Horm Res Paediatr. (2018) 89:372–9. 10.1159/000488468 29886495

[165] Cotella EM Morano RL Wulsin AC Martelle SM Lemen P Fitzgerald M. Lasting impact of chronic adolescent stress and glucocorticoid receptor selective modulation in male and female rats. Psychoneuroendocrinology. (2020) 112:104490. 10.1016/j.psyneuen.2019.104490 31786480 PMC7391799

[166] Midzak A Papadopoulos V. Adrenal mitochondria and steroidogenesis: from individual proteins to functional protein assemblies. Front Endocrinol. (2016) 7:106. 10.3389/fendo.2016.00106 27524977 PMC4965458

[167] Finsterwald C Alberini CM. Stress and glucocorticoid receptor-dependent mechanisms in long-term memory: from adaptive responses to psychopathologies. Neurobiol Learn Mem. (2014) 112:17–29. 10.1016/j.nlm.2013.09.017 24113652 PMC3979509

[168] Prasad R Kowalczyk JC Meimaridou E Storr HL Metherell LA. Oxidative stress and adrenocortical insufficiency. J Endocrinol. (2014) 221:R63–73. 10.1530/JOE-13-0346 24623797 PMC4045218

[169] Jaszczyk A Juszczak GR. Glucocorticoids, metabolism and brain activity. Neurosci Biobehav Rev. (2021) 126:113–45. 10.1016/j.neubiorev.2021.03.007 33727030

[170] Choi GE Han HJ. Glucocorticoid impairs mitochondrial quality control in neurons. Neurobiol Dis. (2021) 152:105301. 10.1016/j.nbd.2021.105301 33609641

[171] Kasahara E Inoue M. Cross-talk between HPA-axis-increased glucocorticoids and mitochondrial stress determines immune responses and clinical manifestations of patients with sepsis. Redox Report. (2015) 20:1–10. 10.1179/1351000214Y.0000000107 25310535 PMC6837532

[172] Kokkinopoulou I Moutsatsou P. Mitochondrial glucocorticoid receptors and their actions. Int J Mol Sci. (2021) 22:6054. 10.3390/ijms22116054 34205227 PMC8200016

[173] Lapp HE Bartlett AA Hunter RG. Stress and glucocorticoid receptor regulation of mitochondrial gene expression. J Mol Endocrinol. (2019) 62:R121–8. 10.1530/JME-18-0152 30082335

[174] Lee SR Kim HK Song IS Youm J Dizon LA Jeong SH. Glucocorticoids and their receptors: insights into specific roles in mitochondria. Prog Biophys Mol Biol. (2013) 112:44–54. 10.1016/j.pbiomolbio.2013.04.001 23603102

[175] Picard M Juster RP McEwen BS. Mitochondrial allostatic load puts the “gluc” back in glucocorticoids. Nat Rev Endocrinol. (2014) 10:303–10. 10.1038/nrendo.2014.22 24663223

[176] Zitkovsky EK Daniels TE Tyrka AR. Mitochondria and early-life adversity. Mitochondrion. (2021) 57:213–21. 10.1016/j.mito.2021.01.005 33484871 PMC8172448

[177] Du J Wang Y Hunter R Wei Y Blumenthal R Falke C. Dynamic regulation of mitochondrial function by glucocorticoids. Proc Natl Acad Sci USA. (2009) 106:3543–8. 10.1073/pnas.0812671106 19202080 PMC2637276

[178] He Y Zhang L Zhu Z Xiao A Yu H Gan X. Blockade of cyclophilin D rescues dexamethasone-induced oxidative stress in gingival tissue. PLoS ONE. (2017) 12:e0173270. 10.1371/journal.pone.0173270 28273124 PMC5342226

[179] Tome ME Lee K Jaramillo MC Briehl MM. Mitochondria are the primary source of the H 2O 2 signal for glucocorticoid-induced apoptosis of lymphoma cells. Exp Ther Med. (2012) 4:237–42. 10.3892/etm.2012.595 22844350 PMC3404723

[180] Choi GE Lee HJ Chae CW Cho JH Jung YH Kim JS. BNIP3L/NIX-mediated mitophagy protects against glucocorticoid-induced synapse defects. Nat Commun. (2021) 12:1–8. 10.1038/s41467-020-20679-y 33473105 PMC7817668

[181] Dempster KS O'Leary DD MacNeil AJ Hodges GJ Wade TJ. Linking the hemodynamic consequences of adverse childhood experiences to an altered HPA axis and acute stress response. Brain Behav Immun. (2021) 93:254–63. 10.1016/j.bbi.2020.12.018 33358983

[182] Picard M McManus MJ Gray JD Nasca C Moffat C Kopinski PK. Mitochondrial functions modulate neuroendocrine, metabolic, inflammatory, and transcriptional responses to acute psychological stress. Proc Natl Acad Sci USA. (2015) 112:E6614–23. 10.1073/pnas.1515733112 26627253 PMC4672794

[183] Gimsa U Kanitz E Otten W Ibrahim SM. Behavior and stress reactivity in mouse strains with mitochondrial DNA variations. Ann N Y Acad Sci. (2009) 1153:131–8. 10.1111/j.1749-6632.2008.03960.x 19236336

[184] Ridout KK Coe JL Parade SH Marsit CJ Kao HT Porton B. Molecular markers of neuroendocrine function and mitochondrial biogenesis associated with early life stress. Psychoneuroendocrinology. (2020) 116:104632. 10.1016/j.psyneuen.2020.104632 32199200 PMC7887859

[185] Tordjman S Anderson GM Kermarrec S Bonnot O Geoffray MM Brailly-Tabard S. Altered circadian patterns of salivary cortisol in low-functioning children and adolescents with autism. Psychoneuroendocrinology. (2014) 50:227–45. 10.1016/j.psyneuen.2014.08.010 25244637

[186] Alagendran K Hitch D Wadley C Stagnitti K. Cortisol responsivity to social play in children with autism: a systematic review. J Occup Ther Sch Early Interv. (2019) 12:427–43. 10.1080/19411243.2019.1604285

[187] Hadwin JA Lee E Kumsta R Cortese S Kovshoff H. Cortisol awakening response in children and adolescents with autism spectrum disorder: a systematic review and meta-analysis. Evid Based Ment Health. (2019) 22:118–24. 10.1136/ebmental-2019-300098 31253603 PMC10270439

[188] Tomarken AJ Han GT Corbett BA. Temporal patterns, heterogeneity, and stability of diurnal cortisol rhythms in children with autism spectrum disorder. Psychoneuroendocrinology. (2015) 62:217–26. 10.1016/j.psyneuen.2015.08.016 26318632 PMC4945957

[189] Corbett BA Schupp CW. The cortisol awakening response (CAR) in male children with autism spectrum disorder. Horm Behav. (2014) 65:345–50. 10.1016/j.yhbeh.2014.01.012 24508619 PMC4004674

[190] Schupp CW Simon D Corbett BA. Cortisol responsivity differences in children with autism spectrum disorders during free and cooperative play. J Autism Dev Disord. (2013) 43:2405–17. 10.1007/s10803-013-1790-2 23430177 PMC3885342

[191] Corbett BA Simon D. Adolescence, stress and cortisol in autism spectrum disorders. OA Autism. (2014) 1:2. 10.13172/2052-7810-1-1-348 24665363 PMC3961758

[192] Makris G Agorastos A Chrousos GP Pervanidou P. Stress system activation in children and adolescents with autism spectrum disorder. Front Neurosci. (2021) 15:756628. 10.3389/fnins.2021.756628 35095389 PMC8793840

[193] Edmiston EK Muscatello RA Corbett BA. Altered pre-ejection period response to social evaluative threat in adolescents with autism spectrum disorder. Res Autism Spectr Disord. (2017) 36:57–65. 10.1016/j.rasd.2017.01.008 29177005 PMC5699479

[194] Muscatello RA Corbett BA. Comparing the effects of age, pubertal development, and symptom profile on cortisol rhythm in children and adolescents with autism spectrum disorder. Autism Research. (2018) 11:110–20. 10.1002/aur.1879 29030905 PMC6453123

[195] Ogawa S Lee YA Yamaguchi Y Shibata Y Goto Y. Associations of acute and chronic stress hormones with cognitive functions in autism spectrum disorder. Neuroscience. (2017) 343:229–39. 10.1016/j.neuroscience.2016.12.003 27956063

[196] Spratt EG Nicholas JS Brady KT Carpenter LA Hatcher CR Meekins KA. Enhanced cortisol response to stress in children in autism. J Autism Dev Disord. (2012) 42:75–81. 10.1007/s10803-011-1214-0 21424864 PMC3245359

[197] Bitsika V Sharpley CF Andronicos NM Agnew LL. Hypothalamus–pituitary–adrenal axis daily fluctuation, anxiety and age interact to predict cortisol concentrations in boys with an autism spectrum disorder. Physiol Behav. (2015) 138:200–7. 10.1016/j.physbeh.2014.11.010 25446203

[198] Bitsika V Sharpley CF McMillan ME Agnew LL. Background cortisol versus social anxiety as correlates of HPA-axis recovery from stress in boys with Autism Spectrum Disorder. Int J Dev Neurosci. (2018) 71:52–60. 10.1016/j.ijdevneu.2018.08.004 30165174

[199] Sharpley CF Bitsika V Andronicos NM Agnew LL. Further evidence of HPA-axis dysregulation and its correlation with depression in Autism Spectrum Disorders: Data from girls. Physiol Behav. (2016) 167:110–7. 10.1016/j.physbeh.2016.09.003 27619171

[200] Patel N Crider A Pandya CD Ahmed AO Pillai A. Altered mRNA levels of glucocorticoid receptor, mineralocorticoid receptor, and co-chaperones (FKBP5 and PTGES3) in the middle frontal gyrus of autism spectrum disorder subjects. Mol Neurobiol. (2016) 53:2090–9. 10.1007/s12035-015-9178-2 25912394

[201] Bozkurt H Çim A Şimşek S Şahin S. Association between the rs1360780 polymorphism in FKBP5 gene and serum cortisol levels in children with autism spectrum disorder. Klin Psikofarmakol Bul. (2016) 26: 10.21203/rs.3.rs-411548/v1

[202] Yang T Liu J Zhang Y Zhang Q Shangguan L Li Z. Coping style predicts sense of security and mediates the relationship between autistic traits and social anxiety: moderation by a polymorphism of the FKBP5 gene. Behav Brain Res. (2021) 404:113142. 10.1016/j.bbr.2021.113142 33508350

[203] Sharpley CF Bitsika V Agnew LL Andronicos NM. Is daily replication necessary when sampling cortisol concentrations in association studies of children with autism spectrum disorder? A systematic review and discussion paper. Rev Neurosci. (2017) 28:103–11. 10.1515/revneuro-2016-0037. [Epub ahead of print]. 27533116

[204] Brown M Worrell C Pariante CM. Inflammation and early life stress: an updated review of childhood trauma and inflammatory markers in adulthood. Pharmacol Biochem Behav. (2021) 211:173291. 10.1016/j.pbb.2021.173291 34695507

[205] Dutcher EG Pama EAC Lynall ME Khan S Clatworthy MR Robbins TW. Early-life stress and inflammation: a systematic review of a key experimental approach in rodents. Brain Neurosci Adv. (2020) 4:2398212820978049. 10.1177/2398212820978049 33447663 PMC7780197

[206] Carloni E Ramos A Hayes LN. Developmental stressors induce innate immune memory in microglia and contribute to disease risk. Int J Mol Sci. (2021) 22:13035. 10.3390/ijms222313035 34884841 PMC8657756

[207] Lurie DI. An integrative approach to neuroinflammation in psychiatric disorders and neuropathic pain. J Exp Neurosci. (2018) 12:1179069518793639. 10.1177/1179069518793639 30127639 PMC6090491

[208] Furtado M Katzman MA. Examining the role of neuroinflammation in major depression. Psychiatry Res. (2015) 229:27–36. 10.1016/j.psychres.2015.06.009 26187338

[209] Troubat R Barone P Leman S Desmidt T Cressant A Atanasova B. Neuroinflammation and depression: a review. Eur J Neurosci. (2021) 53:151–71. 10.1111/ejn.14720 32150310

[210] Bottaccioli AG Bologna M Bottaccioli F. Psychic life-biological molecule bidirectional relationship: pathways, mechanisms, and consequences for medical and psychological sciences — a narrative review. Int J Mol Sci. (2022) 23:3932. 10.3390/ijms23073932 35409300 PMC8999976

[211] Zhu Y Klomparens EA Guo S Geng X. Neuroinflammation caused by mental stress: the effect of chronic restraint stress and acute repeated social defeat stress in mice. Neurol Res. (2019) 41:762–9. 10.1080/01616412.2019.1615670 31092145

[212] Eidson LN deSousa Rodrigues ME Johnson MA Barnum CJ Duke BJ Yang Y. Chronic psychological stress during adolescence induces sex-dependent adulthood inflammation, increased adiposity, and abnormal behaviors that are ameliorated by selective inhibition of soluble tumor necrosis factor with XPro1595. Brain Behav Immun. (2019) 81:305–16. 10.1016/j.bbi.2019.06.027 31251975 PMC8597195

[213] Cao P Chen C Liu A Shan Q Zhu X Jia C. Early-life inflammation promotes depressive symptoms in adolescence via microglial engulfment of dendritic spines. Neuron. (2021) 109:2573–89.e9. 10.1016/j.neuron.2021.06.012 34233151

[214] Bekhbat M Howell PA Rowson SA Kelly SD Tansey MG Neigh GN. Chronic adolescent stress sex-specifically alters central and peripheral neuro-immune reactivity in rats. Brain Behav Immun. (2019) 76:248–57. 10.1016/j.bbi.2018.12.005 30550932 PMC6886374

[215] Catale C Gironda S Iacono L Lo Carola V. Microglial function in the effects of early-life stress on brain and behavioral development. J Clin Med. (2020) 9:468. 10.3390/jcm9020468 32046333 PMC7074320

[216] Petrelli F Pucci L Bezzi P. Astrocytes and microglia and their potential link with autism spectrum disorders. Front Cell Neurosci. (2016) 10:21. 10.3389/fncel.2016.00021 26903806 PMC4751265

[217] Gładysz D Krzywdzińska A Hozyasz KK. Immune abnormalities in autism spectrum disorder—could they hold promise for causative treatment? Mol Neurobiol. (2018) 55:6387–435. 10.1007/s12035-017-0822-x 29307081 PMC6061181

[218] Mekori-Domachevsky E Segal-Gavish H Gross R. Neuroinflammation and neuroprotection in schizophrenia and autism spectrum disorder. In: Neuroprotection in Autism, Schizophrenia and Alzheimer's Disease. Amsterdam: Elsevier (2019). p. 101–22.

[219] Liao X Liu Y Fu X Li Y. Postmortem studies of neuroinflammation in autism spectrum disorder: a systematic review. Mol Neurobiol. (2020) 57:3424–38. 10.1007/s12035-020-01976-5 32529489

[220] Liao X Yang J Wang H Li Y. Microglia mediated neuroinflammation in autism spectrum disorder. J Psychiatr Res. (2020) 130:167–76. 10.1016/j.jpsychires.2020.07.013 32823050

[221] Liao X Li Y. Nuclear factor kappa B in autism spectrum disorder: a systematic review. Pharmacol Res. (2020) 159:104918. 10.1016/j.phrs.2020.104918 32461184

[222] Fatemi SH Folsom TD Reutiman TJ Lee S. Expression of astrocytic markers aquaporin 4 and connexin 43 is altered in brains of subjects with autism. Synapse. (2008) 62:501. 10.1002/syn.20519 18435417 PMC2697054

[223] Wegiel J Kuchna I Nowicki K Imaki H Wegiel J Marchi E. The neuropathology of autism: defects of neurogenesis and neuronal migration, and dysplastic changes. Acta Neuropathol. (2010) 119:755–70. 10.1007/s00401-010-0655-4 20198484 PMC2869041

[224] Tetreault NA Hakeem AY Jiang S Williams BA Allman E Wold BJ. Microglia in the cerebral cortex in autism. J Autism Dev Disord. (2012) 42:2569–84. 10.1007/s10803-012-1513-0 22466688

[225] Suzuki K Sugihara G Ouchi Y Nakamura K Futatsubashi M Takebayashi K. Microglial activation in young adults with autism spectrum disorder. JAMA Psychiatry. (2013) 70:49–58. 10.1001/jamapsychiatry.2013.272 23404112

[226] Edmonson C Ziats MN Rennert OM. Altered glial marker expression in autistic post-mortem prefrontal cortex and cerebellum. Mol Autism. (2014) 5:3. 10.1186/2040-2392-5-3 24410870 PMC3914711

[227] Gupta S Ellis SE Ashar FN Moes A Bader JS Zhan J. Transcriptome analysis reveals dysregulation of innate immune response genes and neuronal activity-dependent genes in autism. Nat Commun. (2014) 5:1–8. 10.1038/ncomms6748 25494366 PMC4270294

[228] Voineagu I Wang X Johnston P Lowe JK Tian Y Horvath S. Transcriptomic analysis of autistic brain reveals convergent molecular pathology. Nature. (2011) 474:380–4. 10.1038/nature10110 21614001 PMC3607626

[229] Laurence JA Fatemi SH. Glial fibrillary acidic protein is elevated in superior frontal, parietal and cerebellar cortices of autistic subjects. Cerebellum. (2005) 4:206–10. 10.1080/14734220500208846 16147953

[230] Morgan JT Chana G Pardo CA Achim C Semendeferi K Buckwalter J. Microglial activation and increased microglial density observed in the dorsolateral prefrontal cortex in autism. Biol Psychiatry. (2010) 68:368–76. 10.1016/j.biopsych.2010.05.024 20674603

[231] Vargas DL Nascimbene C Krishnan C Zimmerman AW Pardo CA. Neuroglial activation and neuroinflammation in the brain of patients with autism. Ann Neurol. (2005) 57:67–81. 10.1002/ana.20315 15546155

[232] Fetit R Hillary RF Price DJ Lawrie SM. The neuropathology of autism: a systematic review of post-mortem studies of autism and related disorders. Neurosci Biobehav Rev. (2021) 129:35–62. 10.1016/j.neubiorev.2021.07.014 34273379

[233] Chiang JJ Lam PH Chen E Miller GE. Psychological stress during childhood and adolescence and its association with inflammation across the lifespan: a critical review and meta-analysis. Psychol Bull. (2022) 148:27–66. 10.1037/bul0000351 PMC11378952 39247904

[234] Bronzuoli MR Facchinetti R Ingrassia D Sarvadio M Schiavi S Steardo L. Neuroglia in the autistic brain: evidence from a preclinical model. Mol Autism. (2018) 9:1–17. 10.1186/s13229-018-0254-0 30603062 PMC6307226

[235] Afridi R Kim JH Rahman MH Suk K. Metabolic regulation of glial phenotypes: implications in neuron–glia interactions and neurological disorders. Front Cell Neurosci. (2020) 14:20. 10.3389/fncel.2020.00020 32116564 PMC7026370

[236] Moulson AJ Squair JW Franklin RJM Tetzlaff W Assinck P. Diversity of reactive astrogliosis in CNS pathology: heterogeneity or plasticity? Front Cell Neurosci. (2021) 15:284. 10.3389/fncel.2021.703810 34381334 PMC8349991

[237] Saab AS Tzvetavona ID Trevisiol A Baltan S Dibaj P Kusch K. Oligodendroglial NMDA receptors regulate glucose import and axonal energy metabolism. Neuron. (2016) 91:119–32. 10.1016/j.neuron.2016.05.016 27292539 PMC9084537

[238] Poitelon Y Kopec AM Belin S. Myelin fat facts: an overview of lipids and fatty acid metabolism. Cells. (2020) 9:812. 10.3390/cells9040812 32230947 PMC7226731

[239] Babetto E Wong KM Beirowski B. A glycolytic shift in Schwann cells supports injured axons. Nat Neurosci. (2020) 23:1215–28. 10.1038/s41593-020-0689-4 32807950 PMC8758250

[240] Liddelow SA Marsh SE Stevens B. Microglia and astrocytes in disease: dynamic duo or partners in crime? Trends Immunol. (2020) 41:820–35. 10.1016/j.it.2020.07.006 32819809

[241] Loving BA Bruce KD. Lipid and lipoprotein metabolism in microglia. Front Physiol. (2020) 11:393. 10.3389/fphys.2020.00393 32411016 PMC7198855

[242] Chausse B Kakimoto PA Kann O. Microglia and lipids: how metabolism controls brain innate immunity. Semin Cell Dev Biol. (2021) 112:137–44. 10.1016/j.semcdb.2020.08.001 32807643

[243] Arcuri C Mecca C Bianchi R Giambanco I Donato R. The pathophysiological role of microglia in dynamic surveillance, phagocytosis and structural remodeling of the developing CNS. Front Mol Neurosci. (2017) 10:191. 10.3389/fnmol.2017.00191 28674485 PMC5474494

[244] Zeidán-Chuliá F Salmina AB Malinovskaya NA Noda M Verkhratsky A Moreira JCF. The glial perspective of autism spectrum disorders. Neurosci Biobehav Rev. (2014) 38:160–72. 10.1016/j.neubiorev.2013.11.008 24300694

[245] Galvez-Contreras AY Zarate-Lopez D Torres-Chavez AL Gonzalez-Perez O. Role of oligodendrocytes and myelin in the pathophysiology of autism spectrum disorder. Brain Sciences. (2020) 10:951. 10.3390/brainsci10120951 33302549 PMC7764453

[246] Strunecka A Blaylock R Patocka J Strunecky O. Immunoexcitotoxicity as the central mechanism of etiopathology and treatment of autism spectrum disorders: a possible role of fluoride and aluminum. Surg Neurol Int. (2018) 9:74. 10.4103/sni.sni_407_17 29721353 PMC5909100

[247] Schafer DP Lehrman EK Kautzman AG Koyama R Mardinly AR Yamasaki R. Microglia sculpt postnatal neural circuits in an activity and complement-dependent manner. Neuron. (2012) 74:691–705. 10.1016/j.neuron.2012.03.026 22632727 PMC3528177

[248] Bernier LP York EM MacVicar BA. Immunometabolism in the brain: how metabolism shapes microglial function. Trends Neurosci. (2020) 43:854–69. 10.1016/j.tins.2020.08.008 32958333

[249] Lauro C Limatola C. Metabolic reprograming of microglia in the regulation of the innate inflammatory response. Front Immunol. (2020) 11:493. 10.3389/fimmu.2020.00493 32265936 PMC7099404

[250] Zengeler KE Lukens JR. Innate immunity at the crossroads of healthy brain maturation and neurodevelopmental disorders. Nat Rev Immunol. (2021) 21:454–68. 10.1038/s41577-020-00487-7 33479477 PMC9213174

[251] Clarke LE Barres BA. Emerging roles of astrocytes in neural circuit development. Nat Rev Neurosci. (2013) 14:311–21. 10.1038/nrn3484 23595014 PMC4431630

[252] Sahlender DA Savtchouk I Volterra A. What do we know about gliotransmitter release from astrocytes? Philos Trans R Soc B Biol Sci. (2014) 369:20130592. 10.1098/rstb.2013.0592 25225086 PMC4173278

[253] Cattane N Vernon AC Borsini A Scassellati C Endres D Capuron L. Preclinical animal models of mental illnesses to translate findings from the bench to the bedside: molecular brain mechanisms and peripheral biomarkers associated to early life stress or immune challenges. Eur Neuropsychopharmacol. (2022) 58:55–79. 10.1016/j.euroneuro.2022.02.002 35235897

[254] Rahman MH Suk K. Mitochondrial dynamics and bioenergetic alteration during inflammatory activation of astrocytes. Front Aging Neurosci. (2020) 12:459. 10.3389/fnagi.2020.614410 33362533 PMC7759744

[255] Pellerin L Pellegri G Bittar PG Charnay Y Bouras C Martin JL. Evidence supporting the existence of an activity-dependent astrocyte-neuron lactate shuttle. Dev Neurosci. (1998) 20:291–9. 10.1159/000017324 9778565

[256] Harry GJ Childers G Giridharan S Hernandes IL. An association between mitochondria and microglia effector function: what do we think we know? Neuroimmunol Neuroinflamm. (2020) 7:150–65. 10.20517/2347-8659.2020.07 32934971 PMC7489447

[257] Fairley LH Wong JH Barron AM. Mitochondrial regulation of microglial immunometabolism in Alzheimer's disease. Front Immunol. (2021) 12:257. 10.3389/fimmu.2021.624538 33717134 PMC7947196

[258] Rose J Brian C Woods J Pappa A Panayiotidis MI Powers R. Mitochondrial dysfunction in glial cells: implications for neuronal homeostasis and survival. Toxicology. (2017) 391:109–15. 10.1016/j.tox.2017.06.011 28655545 PMC5681369

[259] Ye J Jiang Z Chen X Liu M Li J Liu N. Electron transport chain inhibitors induce microglia activation through enhancing mitochondrial reactive oxygen species production. Exp Cell Res. (2016) 340:315–26. 10.1016/j.yexcr.2015.10.026 26511505

[260] Park J Choi H Min JS Park SJ Kim JH Park HJ. Mitochondrial dynamics modulate the expression of pro-inflammatory mediators in microglial cells. J Neurochem. (2013) 127:221–32. 10.1111/jnc.12361 23815397

[261] Ferger AI Campanelli L Reimer V Muth KN Merdian I Ludolph AC. Effects of mitochondrial dysfunction on the immunological properties of microglia. J Neuroinflammation. (2010) 7:1–10. 10.1186/1742-2094-7-45 20701773 PMC2925359

[262] Tang Y Le W. Differential roles of M1 and M2 microglia in neurodegenerative diseases. Mol Neurobiol. (2016) 53:1181–94. 10.1007/s12035-014-9070-5 25598354

[263] Gimeno-Bayón J López-López A Rodríguez MJ Mahy N. Glucose pathways adaptation supports acquisition of activated microglia phenotype. J Neurosci Res. (2014) 92:723–31. 10.1002/jnr.23356 24510633

[264] Voloboueva LA Lee SW Emery JF Palmer TD Giffard RG. Mitochondrial protection attenuates inflammation-induced impairment of neurogenesis *in vitro* and *in vivo*. J Neurosci. (2010) 30:12242–51. 10.1523/JNEUROSCI.1752-10.2010 20844120 PMC2972310

[265] Lively S Schlichter LC. Microglia responses to pro-inflammatory stimuli (LPS, IFNγ+TNFα) and reprogramming by resolving cytokines (IL-4, IL-10). Front Cell Neurosci. (2018) 12:215. 10.3389/fncel.2018.00215 30087595 PMC6066613

[266] Freemerman AJ Johnson AR Sacks GN Milner JJ Kirk EL Troester MA. Metabolic reprogramming of macrophages: glucose transporter 1 (GLUT1)-mediated glucose metabolism drives a proinflammatory phenotype. J Biol Chem. (2014) 289:7884–96. 10.1074/jbc.M113.522037 24492615 PMC3953299

[267] Pace TWW Miller AH. Cytokines and glucocorticoid receptor signaling: relevance to major depression. Ann N Y Acad Sci. (2009) 1179:86. 10.1111/j.1749-6632.2009.04984.x 19906234 PMC3399249

[268] Beurel E Toups M Nemeroff CB. The bidirectional relationship of depression and inflammation: double trouble. Neuron. (2020) 107:234–56. 10.1016/j.neuron.2020.06.002 32553197 PMC7381373

[269] Nettis MA Pariante CM. Is there neuroinflammation in depression? Understanding the link between the brain and the peripheral immune system in depression. Int Rev Neurobiol. (2020) 152:23–40. 10.1016/bs.irn.2019.12.004 32450998

[270] Sugama S Kakinuma Y. Stress and brain immunity: microglial homeostasis through hypothalamus-pituitary-adrenal gland axis and sympathetic nervous system. Brain Behav Immun Health. (2020) 7:100111. 10.1016/j.bbih.2020.100111 34589871 PMC8474505

[271] Ahmad MH Rizvi MA Fatima M Mondal AC. Pathophysiological implications of neuroinflammation mediated HPA axis dysregulation in the prognosis of cancer and depression. Mol Cell Endocrinol. (2021) 520:111093. 10.1016/j.mce.2020.111093 33253761

[272] Heidari A Rostam-Abadi Y Rezaei N. The immune system and autism spectrum disorder: association and therapeutic challenges. Acta Neurobiol Exp. (2021) 81:249–63. 10.21307/ane-2021-023 34672295

[273] García-Juárez M Camacho-Morales A. Defining the role of anti- and pro-inflammatory outcomes of Interleukin-6 in mental health. Neuroscience. (2022) 492:32–46. 10.1016/j.neuroscience.2022.03.020 35439579

[274] Zhu Y Wang Y Yao R Hao T Cao J Huang H. Enhanced neuroinflammation mediated by DNA methylation of the glucocorticoid receptor triggers cognitive dysfunction after sevoflurane anesthesia in adult rats subjected to maternal separation during the neonatal period. J Neuroinflammation. (2017) 14:1–16. 10.1186/s12974-016-0782-5 28086911 PMC5234142

[275] Bekhbat M Mukhara D Dozmorov MG Stansfield JC Benusa SD Hyer MM. Adolescent stress sensitizes the adult neuroimmune transcriptome and leads to sex-specific microglial and behavioral phenotypes. Neuropsychopharmacology. (2021) 46:949–58. 10.1038/s41386-021-00970-2 33558677 PMC8115118

[276] Marón FJM Ferder L Saraví FD Manucha W. Hypertension linked to allostatic load: from psychosocial stress to inflammation and mitochondrial dysfunction. Stress. (2018) 22:169–81. 10.1080/10253890.2018.1542683 30547701

[277] Cherry AD Piantadosi CA. Regulation of mitochondrial biogenesis and its intersection with inflammatory responses. Antioxidants Redox Signal. (2015) 22:965–76. 10.1089/ars.2014.6200 25556935 PMC4390030

[278] González-Pardo H Arias JL Gómez-Lázaro E Taboada IL Conejo NM. Sex-specific effects of early life stress on brain mitochondrial function, monoamine levels and neuroinflammation. Brain Sci. (2020) 10:447. 10.3390/brainsci10070447 32674298 PMC7408325

[279] Oginga FO Magwai T Shangase KB Xulu KR Mpofana T. Early life stress and brain plasticity: from alterations of brain morphology to development of psychopathology. NeuroSci. (2022) 3:104–10. 10.3390/neurosci3010008 PMC11523682 39484671

[280] Beckervordersandforth R. Mitochondrial metabolism-mediated regulation of adult neurogenesis. Brain Plasticity. (2017) 3:73–87. 10.3233/BPL-170044 29765861 PMC5928529

[281] Maffezzini C Calvo-Garrido J Wredenberg A Freyer C. Metabolic regulation of neurodifferentiation in the adult brain. Cell Mol Life Sci. (2020) 77:2483–96. 10.1007/s00018-019-03430-9 31912194 PMC7320050

[282] Iwata R Vanderhaeghen P. Regulatory roles of mitochondria and metabolism in neurogenesis. Curr Opin Neurobiol. (2021) 69:231–40. 10.1016/j.conb.2021.05.003 34171617 PMC8415079

[283] Prozorovski T Schneider R Berndt C Hartung HP Aktas O. Redox-regulated fate of neural stem progenitor cells. Biochimica et Biophysica Acta Gen Subjects. (2015) 1850:1543–54. 10.1016/j.bbagen.2015.01.022 25662818

[284] Meyer N Rinholm JE. Mitochondria in myelinating oligodendrocytes: slow and out of breath? Metabolites. (2021) 11:359. 10.3390/metabo11060359 34198810 PMC8226700

[285] Spaas J van Veggel L Schepers M Tiane A van Horssen J Wilson DM. Oxidative stress and impaired oligodendrocyte precursor cell differentiation in neurological disorders. Cell Mol Life Sci. (2021) 78:4615–37. 10.1007/s00018-021-03802-0 33751149 PMC8195802

[286] Tang G Gudsnuk K Kuo SH Cotrina ML Rosoklija G Sosunov A. Loss of mTOR-dependent macroautophagy causes autistic-like synaptic pruning deficits. Neuron. (2014) 83:1131–43. 10.1016/j.neuron.2014.09.001 25155956 PMC4159743

[287] Trifonova EA Khlebodarova TM Gruntenko NE. Molecular mechanisms of autism as a form of synaptic dysfunction. Russ J Genet Appl Res. (2017) 7:869–77. 10.1134/S2079059717080020

[288] Licausi F Hartman NW. Role of mTOR complexes in neurogenesis. Int J Mol Sci. (2018) 19:1544. 10.3390/ijms19051544 29789464 PMC5983636

[289] Pagani M Barsotti N Bertero A Trakoshis S Ulysse L Locarno A. mTOR-related synaptic pathology causes autism spectrum disorder-associated functional hyperconnectivity. Nat Commun. (2021) 12:1–15. 10.1038/s41467-021-26131-z 34667149 PMC8526836

[290] Guo L Tian J Du H. Mitochondrial dysfunction and synaptic transmission failure in Alzheimer's disease. J Alzheimers Dis. (2017) 57:1071–86. 10.3233/JAD-160702 27662318 PMC5605817

[291] Cai Q Tammineni P. Mitochondrial aspects of synaptic dysfunction in Alzheimer's disease. J Alzheimers Dis. (2017) 57:1087–103. 10.3233/JAD-160726 27767992 PMC5398949

[292] Du H Guo L Yan SS. Synaptic mitochondrial pathology in Alzheimer's disease. Antioxid Redox Signal. (2012) 16:1467–75. 10.1089/ars.2011.4277 21942330 PMC3329948

[293] Li J Spletter ML Johnson DA Wright LS Svendsen CN Johnson JA. Rotenone-induced caspase 9/3-independent and -dependent cell death in undifferentiated and differentiated human neural stem cells. J Neurochem. (2005) 92:462–76. 10.1111/j.1471-4159.2004.02872.x 15659217

[294] Mendoza E Miranda-Barrientos JA Vázquez-Roque RA Morales-Herrera E Ruelas A de la Rosa G. In vivo mitochondrial inhibition alters corticostriatal synaptic function and the modulatory effects of neurotrophins. Neuroscience. (2014) 280:156–70. 10.1016/j.neuroscience.2014.09.018 25241069

[295] Yu Q Du F Douglas JT Yu H Yan SS Yan SF. Mitochondrial dysfunction triggers synaptic deficits via activation of p38 MAP kinase signaling in differentiated Alzheimer's disease trans-mitochondrial cybrid cells. J Alzheimers Dis. (2017) 59:223–39. 10.3233/JAD-170283 28598851 PMC5935489

[296] Wang A Zou X Wu J Ma Q Yuan N Ding F. Early-life stress alters synaptic plasticity and mTOR signaling: correlation with anxiety-like and cognition-related behavior. Front Genet. (2020) 11:590068. 10.3389/fgene.2020.590068 33381149 PMC7767996

[297] Martín-Sánchez A González-Pardo H Alegre-Zurano L Castro-Zavala A López-Taboada I Valverde O. Early-life stress induces emotional and molecular alterations in female mice that are partially reversed by cannabidiol. Prog Neuropsychopharmacol Biol Psychiatry. (2022) 115:110508. 10.1016/j.pnpbp.2021.110508 34973413

[298] Tracey TJ Steyn FJ Wolvetang EJ Ngo ST. Neuronal lipid metabolism: multiple pathways driving functional outcomes in health and disease. Front Mol Neurosci. (2018) 11:10. 10.3389/fnmol.2018.00010 29410613 PMC5787076

[299] Cowan M Petri WA Jr. Microglia: immune regulators of neurodevelopment. Front Immunol. (2018) 9:2576. 10.3389/fimmu.2018.02576 30464763 PMC6234957

[300] Noori R Park D Griffiths JD Bells S Frankland PW Mabbott D. Activity-dependent myelination: a glial mechanism of oscillatory self-organization in large-scale brain networks. Proc Natl Acad Sci USA. (2020) 117:13227. 10.1073/pnas.1916646117 32482855 PMC7306810

[301] Ganesan H Balasubramanian V Iyer M Venugopal A Subramaniam MD Cho SGG. mTOR signalling pathway - a root cause for idiopathic autism? BMB Rep. (2019) 52:424. 10.5483/BMBRep.2019.52.7.137 31186084 PMC6675248

[302] Boksha IS Prokhorova TA Tereshkina EB Savushkina OK Burbaeva GSh. Protein phosphorylation signaling cascades in autism: the role of mTOR pathway. Biochemistry. (2021) 86:577–96. 10.1134/S0006297921050072 33993859

[303] Carroll L Braeutigam S Dawes JM Krsnik Z Kostovic I Coutinho E. Autism spectrum disorders: multiple routes to, and multiple consequences of, abnormal synaptic function and connectivity. Neuroscientist. (2021) 27:10–29. 10.1177/1073858420921378 32441222 PMC7804368

[304] Ohta K-Ichi Suzuki S Warita K Sumitani K Tenkumo C Ozawa T. The effects of early life stress on the excitatory/inhibitory balance of the medial prefrontal cortex. Behav Brain Res. (2020) 379:112306. 10.1016/j.bbr.2019.112306 31629835

[305] Hyer MM Shaw GA Goswamee P Dyer SK Burns CM Soriano E. Chronic adolescent stress causes sustained impairment of cognitive flexibility and hippocampal synaptic strength in female rats. Neurobiol Stress. (2021) 14:100303. 10.1016/j.ynstr.2021.100303 33614865 PMC7876631

[306] Deutschmann AU Kirkland JM Briand LA. Adolescent social isolation induced alterations in nucleus accumbens glutamate signalling. Addict Biol. (2021) 27:e13077. 10.1111/adb.13077 34278652 PMC9206853

[307] Xu H Wang J Jing H Ellenbroek B Shao F Wang W. mPFC GABAergic transmission mediated the role of BDNF signaling in cognitive impairment but not anxiety induced by adolescent social stress. Neuropharmacology. (2021) 184:108412. 10.1016/j.neuropharm.2020.108412 33245959

[308] Wang Q Sun YN Zou CM Zhang TL Li Z Liu M. Regulation of the kynurenine/serotonin pathway by berberine and the underlying effect in the hippocampus of the chronic unpredictable mild stress mice. Behav Brain Res. (2022) 422:113764. 10.1016/j.bbr.2022.113764 35051489

[309] Oswald LM Dunn KE Seminowicz DA Storr CL. Early life stress and risks for opioid misuse: review of data supporting neurobiological underpinnings. J Personal Med. (2021) 11:315. 10.3390/jpm11040315 33921642 PMC8072718

[310] Bonapersona V Joëls M Sarabdjitsingh RA. Effects of early life stress on biochemical indicators of the dopaminergic system: a 3 level meta-analysis of rodent studies. Neurosci Biobehav Rev. (2018) 95:1–16. 10.1016/j.neubiorev.2018.09.003 30201218

[311] Majcher-Maślanka I Solarz A Wedzony K Chocyk A. The effects of early-life stress on dopamine system function in adolescent female rats. Int J Dev Neurosci. (2017) 57:24–33. 10.1016/j.ijdevneu.2017.01.001 28065748

[312] Catale C lo Iacono L Martini A Heil C Guatteo E Mercuri NB. Early life social stress causes sex- and region-dependent dopaminergic changes that are prevented by minocycline. Mol Neurobiol. (2022) 59:3913–32. 10.1007/s12035-022-02830-6 35435618 PMC9148283

[313] Oswald LM Wand GS Kuwabara H Wong DF Zhu S Brasic JR. History of childhood adversity is positively associated with ventral striatal dopamine responses to amphetamine. Psychopharmacology. (2014) 231:2417–33. 10.1007/s00213-013-3407-z 24448898 PMC4040334

[314] Pruessner JC Champagne F Meaney MJ Dagher A. Dopamine release in response to a psychological stress in humans and its relationship to early life maternal care: a positron emission tomography study using [11C]Raclopride. J Neurosci. (2004) 24:2825–31. 10.1523/JNEUROSCI.3422-03.2004 15028776 PMC6729514

[315] Averill LA Abdallah CG Fenton LR Fasula MK Jiang L Rothman DL. Early life stress and glutamate neurotransmission in major depressive disorder. Eur Neuropsychopharmacol. (2020) 35:71–80. 10.1016/j.euroneuro.2020.03.015 32418842 PMC7913468

[316] Underwood MD Kassir SA Bakalian MJ Galfalvy H Dwork AJ Mann JJ. Serotonin receptors and suicide, major depression, alcohol use disorder and reported early life adversity. Transl Psychiatry. (2018) 8:1–15. 10.1038/s41398-018-0309-1 30552318 PMC6294796

[317] Pompili M Serafini G Innamorati M Möller-Leimkühler AM Giupponi G Girardi P. The hypothalamic-pituitary-adrenal axis and serotonin abnormalities: a selective overview for the implications of suicide prevention. Eur Arch Psychiatry Clin Neurosci. (2010) 260:583–600. 10.1007/s00406-010-0108-z 20174927

[318] Lee EJ Choi SY Kim E. NMDA receptor dysfunction in autism spectrum disorders. Curr Opin Pharmacol. (2015) 20:8–13. 10.1016/j.coph.2014.10.007 25636159

[319] Marotta R Risoleo MC Messina G Parisi L Carotenuto M Vetri L. The neurochemistry of autism. Brain Sci. (2020) 10:1–18. 10.3390/brainsci10030163 32182969 PMC7139720

[320] Essa MM Braidy N Vijayan KR Subash S Guillemin GJ. Excitotoxicity in the pathogenesis of autism. Neurotoxicity Res. (2013) 23:393–400. 10.1007/s12640-012-9354-3 23065398

[321] Vieira MM Jeong J Roche KW. The role of NMDA receptor and neuroligin rare variants in synaptic dysfunction underlying neurodevelopmental disorders. Curr Opin Neurobiol. (2021) 69:93–104. 10.1016/j.conb.2021.03.001 33823469

[322] Yang P Chang CL. Glutamate-mediated signaling and autism spectrum disorders: emerging treatment targets. Curr Pharm Des. (2014) 20:5186–93. 10.2174/1381612819666140110120725 24410563

[323] Zheng Z Zhu T Qu Y Mu D. Blood glutamate levels in autism spectrum disorder: a systematic review and meta-analysis. PLoS ONE. (2016) 11:e0158688. 10.1371/journal.pone.0158688 27390857 PMC4938426

[324] Bjørklund G Tinkov AA Hosnedlová B Kizek R Ajsuvakova OP Chirumbolo S. The role of glutathione redox imbalance in autism spectrum disorder: a review. Free Radic Biol Med. (2020) 160:149–62. 10.1016/j.freeradbiomed.2020.07.017 32745763

[325] Kawada K Kuramoto N Mimori S. Possibility that the onset of autism spectrum disorder is induced by failure of the glutamine-glutamate cycle. Curr Mol Pharmacol. (2020) 13. 10.2174/1874467213666200319125109 32189600

[326] László A Horváth E Eck E Fekete M. Serum serotonin, lactate and pyruvate levels in infantile autistic children. Clin Chim Acta. (1994) 229:205–7. 10.1016/0009-8981(94)90243-7 7988051

[327] Pagan C Delorme R Callebert J Goubran-Botros H Amsellem F Drouot X. The serotonin-N-acetylserotonin-melatonin pathway as a biomarker for autism spectrum disorders. Transl Psychiatry. (2014) 4:e479. 10.1038/tp.2014.120 25386956 PMC4259991

[328] Aaron E Montgomery A Ren X Guter S Anderson G Carneiro AMD. Whole blood serotonin levels and platelet 5-HT 2A binding in autism spectrum disorder. J Autism Dev Disord. (2019) 49:2417–25. 10.1007/s10803-019-03989-z 30927179 PMC6549243

[329] Muller CL Anacker AMJ Veenstra-VanderWeele J. The serotonin system in autism spectrum disorder: from biomarker to animal models. Neuroscience. (2016) 321:24–41. 10.1016/j.neuroscience.2015.11.010 26577932 PMC4824539

[330] Daly E Tricklebank MD Wichers R. Neurodevelopmental roles and the serotonin hypothesis of autism spectrum disorder. In: The Serotonin System. Amsterdam: Elsevier (2019). p. 23–44.

[331] Pavăl D. A dopamine hypothesis of autism spectrum disorder. Dev Neurosci. (2017) 39:355–60. 10.1159/000478725 28750400

[332] DiCarlo GE Aguilar JI Matthies HJG Harrison FE Bundschuh KE West A. Autism-linked dopamine transporter mutation alters striatal dopamine neurotransmission and dopamine-dependent behaviors. J Clin Investig. (2019) 129:3407–19. 10.1172/JCI127411 31094705 PMC6668686

[333] Bandelow B Bandelow B. Current and novel psychopharmacological drugs for anxiety disorders. Adv Exp Med Biol. (2020) 1191:347–65. 10.1007/978-981-32-9705-0_19 32002937

[334] Wieronska JM Stachowicz K Nowak G Pilc A. The loss of glutamate-GABA harmony in anxiety disorders. In: Kalinin V, editor. Anxiety Disorders. London. In Tech Open (2011). p. 135–58. 10.5772/19919

[335] Ramos-Prats A Kölldorfer J Paolo E Zeidler M Schmid G Ferraguti F. An appraisal of the influence of the metabotropic glutamate 5 (mglu5) receptor on sociability and anxiety. Front Mol Neurosci. (2019) 12:30. 10.3389/fnmol.2019.00030 30873001 PMC6401637

[336] Dogra S Conn PJ. Targeting metabotropic glutamate receptors for the treatment of depression and other stress-related disorders. Neuropharmacology. (2021) 196:108687. 10.1016/j.neuropharm.2021.108687 34175327 PMC8435015

[337] Murray SL Holton KF. Post-traumatic stress disorder may set the neurobiological stage for eating disorders: a focus on glutamatergic dysfunction. Appetite. (2021) 167:105599. 10.1016/j.appet.2021.105599 34271078

[338] Mihov Y Treyer V Akkus F Toman E Milos G Ametamey SM. Metabotropic glutamate receptor 5 in bulimia nervosa. Sci Rep. (2020) 10:1–10. 10.1038/s41598-020-63389-7 32286451 PMC7156702

[339] Oliveira TPD Gonçalves BDC Oliveira BS de Oliveira ACP Reis HJ Ferreira CN. Negative modulation of the metabotropic glutamate receptor type 5 as a potential therapeutic strategy in obesity and binge-like eating behavior. Front Neurosci. (2021) 15:631311. 10.3389/fnins.2021.631311 33642987 PMC7902877

[340] Hermens DF Simcock G Dutton M Bouças AP Can AT Lilley C. Anorexia nervosa, zinc deficiency and the glutamate system: the ketamine option. Prog Neuropsychopharmacol Biol Psychiatry. (2020) 101:109921. 10.1016/j.pnpbp.2020.109921 32169564

[341] Karthik S Sharma LP Narayanaswamy JC. Investigating the role of glutamate in obsessive-compulsive disorder: current perspectives. Neuropsychiatr Dis Treat. (2020) 16:1003. 10.2147/NDT.S211703 32368062 PMC7173854

[342] Martinotti G Chiappini S Pettorruso M Mosca A Miuli A Di Carlo F. Therapeutic potentials of ketamine and esketamine in obsessive–compulsive disorder (OCD), substance use disorders (SUD) and eating disorders (ED): a review of the current literature. Brain Sci. (2021) 11:856. 10.3390/brainsci11070856 34199023 PMC8301752

[343] Roberts-Wolfe D Kalivas P. Glutamate transporter GLT-1 as a therapeutic target for substance use disorders. CNS Neurol Disord Drug Targets. (2015) 14:745–56. 10.2174/1871527314666150529144655 26022265 PMC4730383

[344] Liu Y Zhao J Guo W. Emotional roles of mono-aminergic neurotransmitters in major depressive disorder and anxiety disorders. Front Psychol. (2018) 9:2201. 10.3389/fpsyg.2018.02201 30524332 PMC6262356

[345] Daut RA Fonken LK. Circadian regulation of depression: a role for serotonin. Front Neuroendocrinol. (2019) 54:100746. 10.1016/j.yfrne.2019.04.003 31002895 PMC9826732

[346] Belujon P Grace AA. Dopamine system dysregulation in major depressive disorders. Int J Neuropsychopharmacol. (2017) 20:1036–46. 10.1093/ijnp/pyx056 29106542 PMC5716179

[347] Floris G Cadeddu R Bortolato M. The effects of serotonin degradation on psychopathology: role of monoamine oxidase. Handb Behav Neurosci. (2020) 31:267–78. 10.1016/B978-0-444-64125-0.00014-1

[348] Gosmann NP De Abreu Costa M De Barros Jaeger M Motta LS Frozi J Spanemberg L. Selective serotonin reuptake inhibitors, and serotonin and norepinephrine reuptake inhibitors for anxiety, obsessive-compulsive, and stress disorders: a 3-level network meta-analysis. PLoS Med. (2021) 18:e1003664. 10.1371/journal.pmed.1003664 34111122 PMC8224914

[349] Hou Y-Wei Xiong P Gu X Huang X Wang M Wu J. Association of serotonin receptors with attention deficit hyperactivity disorder: a systematic review and meta-analysis. Curr Med Sci. (2018) 38:538–51. 10.1007/s11596-018-1912-3 30074224

[350] Mills JG Thomas SJ Larkin TA Deng C. Overeating and food addiction in major depressive disorder: links to peripheral dopamine. Appetite. (2020) 148:104586. 10.1016/j.appet.2020.104586 31926176

[351] Beeler JA Burghardt NS. Commentary on vulnerability and resilience to activity-based anorexia and the role of dopamine. J Exp Neurol. (2021) 2:21. 10.33696/Neurol.2.031 33768216 PMC7990270

[352] Gunther KE Pérez-Edgar K. Dopaminergic associations between behavioral inhibition, executive functioning, and anxiety in development. Dev Rev. (2021) 60:100966. 10.1016/j.dr.2021.100966

[353] Inozemtseva O Mejía Núñez E. Executive dysfunction associated with substance abuse. In: Ardila A Fatima S Rosselli M editors. Dysexecutive Syndromes. Cham: Springer (2019). p. 123–42. 10.1007/978-3-030-25077-5_6

[354] Berardelli I Serafini G Cortese N Fiaschè F O'connor RC Pompili M. The involvement of hypothalamus–pituitary–adrenal (HPA) axis in suicide risk. Brain Sci. (2020) 10:653. 10.3390/brainsci10090653 32967089 PMC7565104

[355] Evanson NK Herman JP. Role of paraventricular nucleus glutamate signaling in regulation of HPA axis stress responses. Interdiscip Inf Sci. (2015) 21:253–60. 10.4036/iis.2015.B.10 26472933 PMC4603839

[356] Mathew SJ Coplan JD Smith ELP Schoepp DD Rosenblum LA Gorman JM. Glutamate—hypothalamic-pituitary-adrenal axis interactions: implications for mood and anxiety disorders. CNS Spectr. (2001) 6:555–64. 10.1017/S1092852900002091 15573019

[357] Herman JP Mueller NK Figueiredo H. Role of GABA and glutamate circuitry in hypothalamo-pituitary-adrenocortical stress integration. Ann N Y Acad Sci. (2004) 1018:35–45. 10.1196/annals.1296.004 15240350

[358] Gulyaeva NV. Glucocorticoid regulation of the glutamatergic synapse: mechanisms of stress-dependent neuroplasticity. J Evolut Biochem Physiol. (2021) 57:564–76. 10.1134/S0022093021030091

[359] Sbrini G Brivio P Peeva PM Todiras M Bader M Alenina N. The absence of serotonin in the brain alters acute stress responsiveness by interfering with the genomic function of the glucocorticoid receptors. Front Cell Neurosci. (2020) 14:128. 10.3389/fncel.2020.00128 32547368 PMC7278285

[360] Grino M Guillaume V Caslanas E Boudouresque F Conte-Devolx B Oliver C. Effect of passive immunization against corticotropin-releasing factor (CRF) on the postadrenalectomy changes of CRF binding sites in the rat anterior pituitary gland. Neuroendocrinology. (1987) 45:492–7. 10.1159/000124780 3039393

[361] López JF Chalmers DT Little KY Watson SJ. Regulation of serotonin(1A), glucocorticoid, and mineralocorticoid receptor in rat and human hippocampus: implications for the neurobiology of depression. Biol Psychiatry. (1998) 43:547–73. 10.1016/S0006-3223(97)00484-8 9564441

[362] Oswald LM Wong DF McCaul M Zhou Y Kuwabara H Choi L. Relationships among ventral striatal dopamine release, cortisol secretion, and subjective responses to amphetamine. Neuropsychopharmacology. (2005) 30:821–32. 10.1038/sj.npp.1300667 15702139

[363] DeRosse P Barber AD. Overlapping neurobiological substrates for early-life stress and resilience to psychosis. Biol Psychiatry Cogn Neurosci Neuroimaging. (2021) 6:144–53. 10.1016/j.bpsc.2020.09.003 33097471 PMC7878198

[364] Lopez SA Flagel SB. A proposed role for glucocorticoids in mediating dopamine-dependent cue-reward learning. Stress. (2020) 24:154–67. 10.1080/10253890.2020.1768240 32396486 PMC7728625

[365] Cavalcanti-de-Albuquerque JP de-Souza-Ferreira E de Carvalho DP Galina A. Coupling of GABA metabolism to mitochondrial glucose phosphorylation. Neurochem Res. (2021) 47:470–80. 10.1007/s11064-021-03463-2 34623563

[366] Aldana BI Zhang Y Jensen P Chandrasekaran A Christensen SK Nielsen TT. Glutamate-glutamine homeostasis is perturbed in neurons and astrocytes derived from patient iPSC models of frontotemporal dementia. Mol Brain. (2020) 13:125. 10.1186/s13041-020-00658-6 32928252 PMC7491073

[367] Johnson SC Kayser EB Bornstein R Stokes J Bitto A Park KY. Regional metabolic signatures in the Ndufs4(KO) mouse brain implicate defective glutamate/α-ketoglutarate metabolism in mitochondrial disease. Mol Genet Metab. (2020) 130:118–32. 10.1016/j.ymgme.2020.03.007 32331968 PMC7272141

[368] Gardner A Boles RG. Beyond the serotonin hypothesis: mitochondria, inflammation and neurodegeneration in major depression and affective spectrum disorders. Prog Neuropsychopharmacol Biol Psychiatry. (2011) 35:730–43. 10.1016/j.pnpbp.2010.07.030 20691744

[369] de la Fuente C Burke DG Eaton S Heales SJR. Inhibition of neuronal mitochondrial complex I or lysosomal glucocerebrosidase is associated with increased dopamine and serotonin turnover. Neurochem Int. (2017) 109:94–100. 10.1016/j.neuint.2017.02.013 28242245

[370] Fanibunda SE Deb S Maniyadath B Tiwari P Ghai U Gupta S. Serotonin regulates mitochondrial biogenesis and function in rodent cortical neurons via the 5-HT2A receptor and SIRT1–PGC-1α axis. Proc Natl Acad Sci USA. (2019) 166:11028–37. 10.1073/pnas.1821332116 31072928 PMC6561197

[371] Sterky FH Hoffman AF Milenkovic D Bao B Paganelli A Edgar D. Altered dopamine metabolism and increased vulnerability to MPTP in mice with partial deficiency of mitochondrial complex I in dopamine neurons. Hum Mol Genet. (2012) 21:1078–89. 10.1093/hmg/ddr537 22090423 PMC3277308

[372] Panes J Wendt A Ramirez-Molina O Castro P Fuentealba J. Deciphering the role of PGC-1 in neurological disorders: from mitochondrial dysfunction to synaptic failure. Neural Regen Res. (2022) 17:237–45. 10.4103/1673-5374.317957 34269182 PMC8463972

[373] Ben-Shachar D. The bimodal mechanism of interaction between dopamine and mitochondria as reflected in Parkinson's disease and in schizophrenia. J Neural Transm. (2020) 127:159–68. 10.1007/s00702-019-02120-x 31848775

[374] Brenner-Lavie H Klein E Zuk R Gazawi H Ljubuncic P Ben-Shachar D. Dopamine modulates mitochondrial function in viable SH-SY5Y cells possibly via its interaction with complex I: relevance to dopamine pathology in schizophrenia. Biochim Biophys Acta Bioenerg. (2008) 1777:173–85. 10.1016/j.bbabio.2007.10.006 17996721

[375] Burbulla LF Song P Mazzulli JR Zampese E Wong YC Jeon S. Dopamine oxidation mediates mitochondrial and lysosomal dysfunction in Parkinson's disease. Science. (2017) 357:1255–61. 10.1126/science.aam9080 28882997 PMC6021018

[376] Patel AB De Graaf RA Mason GF Kanamatsu T Rothman DL Shulman RG. Glutamatergic neurotransmission and neuronal glucose oxidation are coupled during intense neuronal activation. J Cerebral Blood Flow Metabolism. (2004) 24:972–85. 10.1097/01.WCB.0000126234.16188.71 15356418

[377] Sonnay S Poirot J Just N Clerc AC Gruetter R Rainer G. Astrocytic and neuronal oxidative metabolism are coupled to the rate of glutamate–glutamine cycle in the tree shrew visual cortex. Glia. (2018) 66:477–91. 10.1002/glia.23259 29120073

[378] Robinson MB Lee ML DaSilva S. Glutamate transporters and mitochondria: signaling, co-compartmentalization, functional coupling, and future directions. Neurochem Res. (2020) 45:526–40. 10.1007/s11064-020-02974-8 32002773 PMC7060825

[379] Kiemes A Davies C Kempton MJ Lukow PB Bennallick C Stone JM. GABA, glutamate and neural activity: a systematic review with meta-analysis of multimodal 1H-MRS-fMRI studies. Front Psychiatry. (2021) 12:255. 10.3389/fpsyt.2021.644315 33762983 PMC7982484

[380] McKenna MC. The glutamate-glutamine cycle is not stoichiometric: fates of glutamate in brain. J Neurosci Res. (2007) 85:3347–58. 10.1002/jnr.21444 17847118

[381] Day PE Matata B Elahic M. Re-visiting glutamate toxicity: implications of monosodium glutamate consumption on glutamate metabolism and metabolic syndrome. J Endocrinol Diabetes Mellit. (2015) 3:20–31. 10.12970/2310-9971.2015.03.01.5

[382] Wong KY Roy J Fung ML Heng BC Zhang C Lim LW. Relationships between mitochondrial dysfunction and neurotransmission failure in Alzheimer's disease. Aging Dis. (2020) 11:1291–316. 10.14336/AD.2019.1125 33014538 PMC7505271

[383] Mosienko V Teschemacher AG Kasparov S. Is L-lactate a novel signaling molecule in the brain? J Cerebral Blood Flow Metabolism. (2015) 35:1069–75. 10.1038/jcbfm.2015.77 25920953 PMC4640281

[384] Michelhaugh SK Guastella AR Mittal S. Overview of the kynurenine pathway of tryptophan metabolism. In: Mittal S editor. Targeting the Broadly Pathogenic Kynurenine Pathway. Cham: Springer (2015). p. 3–9. 10.1007/978-3-319-11870-3_1

[385] Scholpa NE Lynn MK Corum D Boger HA Schnellmann RG. 5-HT1F receptor-mediated mitochondrial biogenesis for the treatment of Parkinson's disease. Br J Pharmacol. (2018) 175:348–58. 10.1111/bph.14076 29057453 PMC5758398

[386] Weng R Shen S Burton C Yang L Nie H Tian Y. Lipidomic profiling of tryptophan hydroxylase 2 knockout mice reveals novel lipid biomarkers associated with serotonin deficiency. Anal Bioanal Chem. (2016) 408:2963–73. 10.1007/s00216-015-9256-3 26780709

[387] Weng R Shen S Tian Y Burton C Xu X Liu Y. Metabolomics approach reveals integrated metabolic network associated with serotonin deficiency. Sci Rep. (2015) 5:1–13. 10.1038/srep11864 26154191 PMC4495385

[388] Molina-Carballo A Jerez-Calero AE Muñoz-Hoyos A. Possible protective role of melatonin in pediatric infectious diseases and neurodevelopmental pathologies. J Child Sci. (2020) 10:e104–9. 10.1055/s-0040-1716713

[389] Li Y Hu N Yang D Oxenkrug G Yang Q. Regulating the balance between the kynurenine and serotonin pathways of tryptophan metabolism. FEBS J. (2017) 284:948–66. 10.1111/febs.14026 28118532

[390] Xie N Zhang L Gao W Huang C Huber PE Zhou X. NAD+ metabolism: pathophysiologic mechanisms and therapeutic potential. Signal Transd Targeted Therapy. (2020) 5:1–37. 10.1038/s41392-020-00311-7 33028824 PMC7539288

[391] Szalardy L Klivenyi P Zadori D Fulop F Toldi J Vecsei L. Mitochondrial disturbances, tryptophan metabolites and neurodegeneration: medicinal chemistry aspects. Curr Med Chem. (2012) 19:1899–920. 10.2174/092986712800167365 22429096

[392] Castro-Portuguez R Sutphin GL. Kynurenine pathway, NAD+ synthesis, and mitochondrial function: targeting tryptophan metabolism to promote longevity and healthspan. Exp Gerontol. (2020) 132:110841. 10.1016/j.exger.2020.110841 31954874 PMC7053056

[393] Savino R Carotenuto M Polito AN Di Noia S Albenzio M Scarinci A. Analyzing the potential biological determinants of autism spectrum disorder: from neuroinflammation to the kynurenine pathway. Brain Sci. (2020) 10:631. 10.3390/brainsci10090631 32932826 PMC7563403

[394] Sigfusdottir ID Kristjansson AL Thorlindsson T Allegrante JP. Stress and adolescent well-being: the need for an interdisciplinary framework. Health Promot Int. (2017) 32:1081–90. 10.1093/heapro/daw038 27153917 PMC5914452

